# Multifunctional magnetic iron oxide nanoparticles: an advanced platform for cancer theranostics

**DOI:** 10.7150/thno.42564

**Published:** 2020-05-15

**Authors:** Shengzhe Zhao, Xujiang Yu, Yuna Qian, Wei Chen, Jianliang Shen

**Affiliations:** 1 State Key Laboratory of Ophthalmology, Optometry and Vision Science, School of Ophthalmology & Optometry, School of Biomedical Engineering, Wenzhou Medical University, Wenzhou 325027, China; 2 Wenzhou Institute, University of Chinese Academy of Sciences, Wenzhou 32500, China.; 3 State Key Lab of Metal Matrix Composites, School of Chemistry and Chemical Engineering, Shanghai Jiao Tong University, Shanghai 200240, China.

**Keywords:** magnetic iron oxide nanoparticles, multifunctional nanoplatform, multimodal imaging, drug delivery, cancer diagnosis and treatment

## Abstract

Multifunctional magnetic nanoparticles and derivative nanocomposites have aroused great concern for multimode imaging and cancer synergistic therapies in recent years. Among the rest, functional magnetic iron oxide nanoparticles (Fe_3_O_4_ NPs) have shown great potential as an advanced platform because of their inherent magnetic resonance imaging (MRI), biocatalytic activity (nanozyme), magnetic hyperthermia treatment (MHT), photo-responsive therapy and drug delivery for chemotherapy and gene therapy. Magnetic Fe_3_O_4_ NPs can be synthesized through several methods and easily surface modified with biocompatible materials or active targeting moieties. The MRI capacity could be appropriately modulated to induce response between *T*_1_ and *T*_2_ modes by controlling the size distribution of Fe_3_O_4_ NPs. Besides, small-size nanoparticles are also desired due to the enhanced permeation and retention (EPR) effect, thus the imaging and therapeutic efficiency of Fe_3_O_4_ NP-based platforms can be further improved. Here, we firstly retrospect the typical synthesis and surface modification methods of magnetic Fe_3_O_4_ NPs. Then, the latest biomedical application including responsive MRI, multimodal imaging, nanozyme, MHT, photo-responsive therapy and drug delivery, the mechanism of corresponding treatments and cooperation therapeutics of multifunctional Fe_3_O_4_ NPs are also be explained. Finally, we also outline a brief discussion and perspective on the possibility of further clinical translations of these multifunctional nanomaterials. This review would provide a comprehensive reference for readers to understand the multifunctional Fe_3_O_4_ NPs in cancer diagnosis and treatment.

## Introduction

Cancer is a class of diseases marked by the malignant proliferation of tumor cells. Despite of the huge advancements achieved during the past decades, cancer still a serious public health issue, which leading the main deaths around the world every year 1. With the development of nanotechnology, multifunctional nanoparticle-based systems have been emerged as a new approach for efficient cancer diagnosis and treatment due to their inherent advantages in overcoming the deficiencies compared to the traditional cancer diagnostic and therapeutic techniques, such as low efficacy, drug resistance or other side effects 2-5. Currently, various multifunctional nanoparticle-based systems integrating nanotechnology and molecular biology together have been developed as powerful tools for cancer early-stage diagnosis, real-time imaging and precise therapy 6-10. The integration of single functional component within a multifunctional therapeutic system has been demonstrated as one effective treatment approach for fighting the cancer 11-13. The ideal nanoparticle-based diagnosis and treatment systems should not only contain the unique physical, chemical and medical properties of nanoparticles, but also be smart carriers to effectively deliver anticancer agents, thus realizing multi-modal imaging and combined therapeutic effects 14-17. Moreover, the diverse nanostructures and surface modification of nanomaterials should endow themselves with the tumor-targeting ability by EPR effect or other interactions affiliated to the features of tumor tissues 18-21.

Magnetic iron oxide nanoparticles (Fe_3_O_4_ NPs) are one representative candidate of multifunctional nanomaterials with increasing utilization in many biomedical fields, including magnetic resonance imaging (MRI), biological catalysis, magnetic hyperthermia, magnetic targeting, magnetic separation, photo-responsive therapy and drug-delivery, and currently have been widely used in tumor diagnosis and treatment 22-28. Meanwhile, according to the specific treatment demand and characteristics of the tumor microenvironment, decoration of the Fe_3_O_4_ NPs with different functionalization factors can form the multifunctional iron oxide nanoparticles to achieve better therapeutic effects 29-31. Compared to other imaging modalities in clinic, MRI exhibits high spatial resolution with rapid* in vivo* image acquisition 32, 33. To improve its sensitivity, MRI contrast agents are usually used, which can be divided into two types, namely *T*_1_ and *T*_2_ agents depending on their unique effects to alter the longitudinal or transverse relaxation time of water protons 34-36. Compared to the widely used *T*_1_-weighted MRI agents of gadolinium (Gd)-based contrast agents with shortcoming of unpredictable renal toxicity and blood circulation time, Fe_3_O_4_ NPs are contrast agents that can be used for either* T*_1_ or *T*_2_-weighted imaging with better biosecurity 13, 37-39. In particular, Fe_3_O_4_ NPs is a typical *T*_2_ contrast agent, when the size of Fe_3_O_4_ less than 5 nm, the decreased magnetic moment will strongly suppressed *T*_2_ effect, thus they can also be used for *T*_1_-weighted MRI imaging, which provides a possibility to prepare responsive *T*_2_-*T*_1_ switching MRI contrast agents 40, 41. Due to the excellent thermal effect under the oscillating magnetic field, magnetic Fe_3_O_4_ NPs are widely used in the field of magnetic hyperthermia treatment of tumors 42. Besides, Fe_3_O_4_ NPs also show catalytic activity analogous peroxidase and considered as mimic enzyme for cancer therapy through the well-known Fenton reactions, which could catalyst endogenous hydrogen peroxide (H_2_O_2_) into the hydroxyl radical (•OH) with high cytotoxicity and cause the death of tumor cells 43, 44. This newly defined chemodynamic therapy (CDT) based on the Fenton reaction is a rapidly developing research topic in cancer treatment these years and have endowed magnetic Fe_3_O_4_ NPs with a brand-new life 45.

The modification of functional components onto Fe_3_O_4_ NPs can also bring photo-responsive therapy, namely photothermal therapy or photodynamic therapy, and makes Fe_3_O_4_ NPs functionalize as advanced nanoplatforms for oncotherapy 46. For example, the modification of magnetic Fe_3_O_4_ NPs with the classic photothermal treatment agent of Au NPs can bring additional photothermal therapeutic capability besides to the MRI and magnetic targeting functionality, thus improving the accuracy of diagnosis and treatment of tumor using Fe_3_O_4_ NPs 47. Similar, Fe_3_O_4_ NPs also have other advantages, such as prolonged blood circulation, fast clearance, low side-effects, excellent imaging and therapeutic efficiency. Therefore, several types of magnetic Fe_3_O_4_-based NPs systems have been approved to translate from experimental stages to clinical applications by the food and drug administration (FDA), some of them have reached the market for clinic (Table 1) 48, 49. For example, the contrast agent AMI-25 (Ferumoxide, Feridex IV, Endorem) consists of the dextran coated Fe_3_O_4_ nanocrystal has been used in clinic, this agent could rapidly agglomeration in liver and spleen after injection 1 h, and the optimal imaging effects will appear in liver and spleen after injection 2 h and 4 h, respectively.

To improve the efficacy of Fe_3_O_4_ NPs in tumor imaging and therapeutic, as well as other inorganic nanomaterials, a maximum tumor accumulation is highly desired 50, 51. Besides to the passive EPR effect 52, 53, the more positive method to increase tumor accumulation of nanomaterials is to conjugation tumor-homing or tumor microenvironment (TME) responsive ligands onto the Fe_3_O_4_ NPs to purposively improve their final amount in tumor tissues through the selective recognition and internalization by tumor cells 54-57. Synthesized Fe_3_O_4_ NPs through common chemical methods are usually coated with the hydrophobic alkyl ligands on the outer surface. Surface modification and further functionalization are necessary to convert them into hydrophilic, improve their biocompatibility and blood circulation time for enhancing tumor accumulation 13, 58, which are also beneficial to reduce the unexpected damage to normal tissues.

Smart drug-delivery systems based on nanoparticles is another vital way to combine chemotherapy with nanotheranostics 59, 60. Fe_3_O_4_ NPs can easily be functionalized into a multifunctional platform by modifying with various therapeutic agents, and the common functional components for surface modification can be classified as: (1) small molecule (e.g., carboxylates, oleic acid) 61, 62; (2) biomolecules (nucleic acids (siRNA), peptides and proteins) 63-65; (3) inorganic materials (e.g., Au, Ag, MoS_2_) 25, 66, 67; (4) mesoporous materials (e.g., mesoporous silica, metal-organic frameworks) 68, 69; (5) polymers (e.g., polyvinylpyrrolidone (PVP), polyethylene glycol (PEG), polyethyleneimine (PEI)) 70-72. Surface modification with these components of nanoparticles usually have a low cytotoxicity, hydrophilic, large pore volume, high surface area, adjustable pore size, in consequence provides the nanoparticles with better biocompatible and biodegradable 73, 74. Based on these, through surface modification and functionalization increases the ability of Fe_3_O_4_ NPs in tumor targeting and responsiveness that further enhance their therapeutic effect 75.

This review aims to summarize the recent progress to provide a clear explanation for the rational design, construction and applications of Fe_3_O_4_ NPs-based multifunctional platform. Firstly, we introduce the common synthesis methods. Secondly, the surface modification and targeting ligand conjugation of Fe_3_O_4_ NPs are described. Finally, we will focus on the recent development of multifunctional Fe_3_O_4_ platforms for cancer diagnosis and treatment, including responsive imaging, nanozyme, CDT, magnetic hyperthermia (MHT), photothermal therapy (PTT), photodynamic therapy (PDT) and drug/gene delivery. We believe the summary of recent development will provide a comprehensive understanding of the applications of Fe_3_O_4_-based NPs multifunctional systems in biomedical research studies and clinic uses.

## Synthesis of magnetic Fe_3_O_4_ NPs

The properties of magnetic Fe_3_O_4_ NPs are initially determined by their size and morphology. Thus, the selection of suitable synthesis method is important. Fe_3_O_4_ NPs can be synthesized by physical, biosynthetic and chemical methods 76-78. Generally, the physical synthesis methods, mainly including the ball grinding method, electron beam lithography, aerosol and gas phase deposition, fail to regulate the size into the nanoscale 79-81. Biosynthetic methods to synthesize magnetic nanoparticles is an emerging technology, which generates magnetosomes through the regulation of biological macromolecules and iron element by magnetotactic bacteria or cells 82, 83. However, these methods are always accompanied with the disadvantages of long synthesis time, low yield and broad size distributions. In comparison, the chemical synthesis methods are relatively common because of the advantages of simple operation, low cost and huge production yields. Therefore, we will only focus on the chemical methods in the main text.

**Figure 1:**
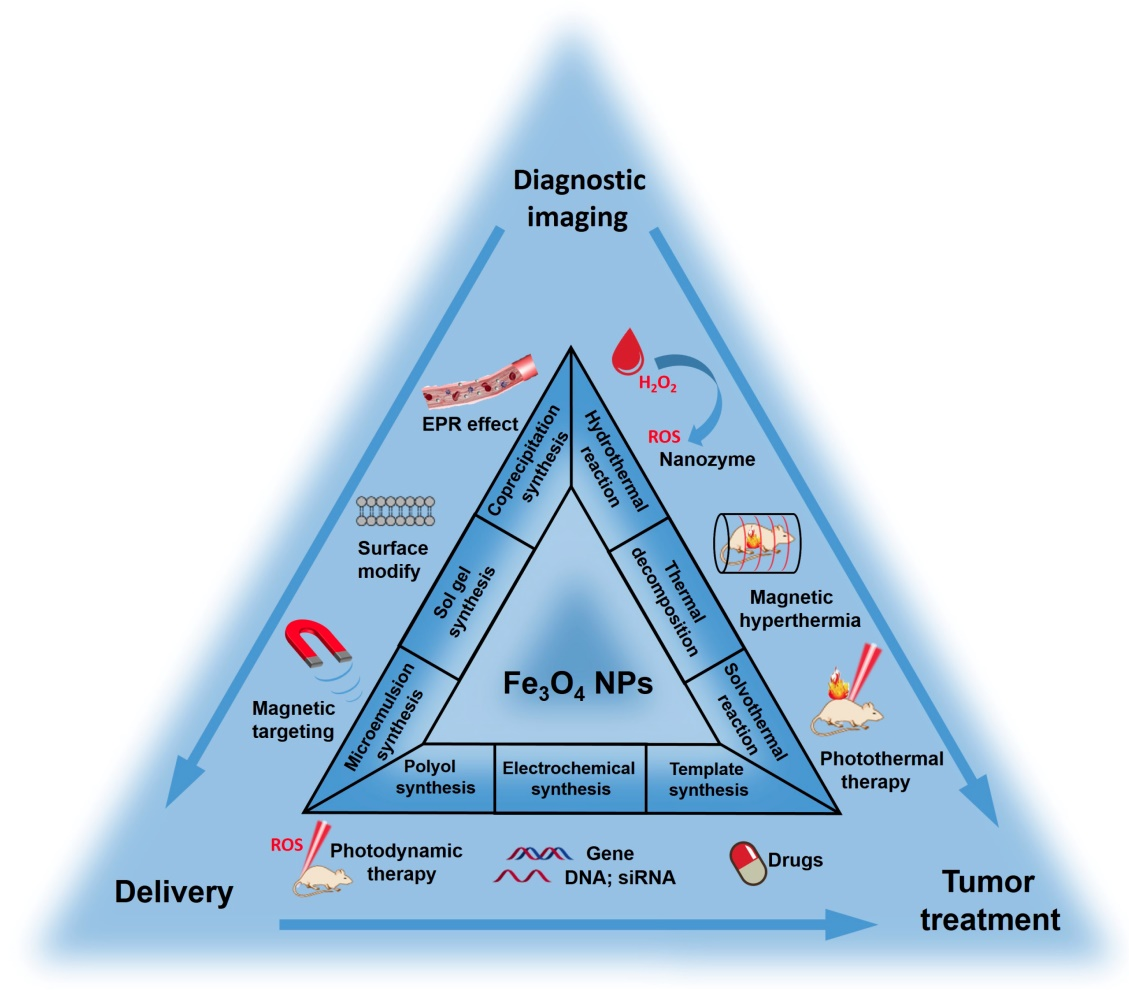
Typical synthesis methods of magnetic Fe_3_O_4_ NPs and their applications in cancer diagnosis and treatment.

In general, the key to synthesize monodisperse Fe_3_O_4_ NPs in solution is to control the processes of nucleation and growth 84, 85. Different synthetic strategies produce Fe_3_O_4_ NPs usual with different morphology, sizes, crystallinity and surface properties 86, 87. The common chemical synthesis methods include thermal decomposition 88, 89, solvothermal reaction 90, polyol synthesis 91, hydrothermal reaction 92, coprecipitation synthesis 93, 94, microemulsion synthesis 95, sol-gel synthesis 96, and template synthesis 97 (Figure 1). Here we will detailly introduce the recent advances using the above methods for the synthesis of Fe_3_O_4_ NPs (Table 2).

### Thermal decomposition

**Figure 2:**
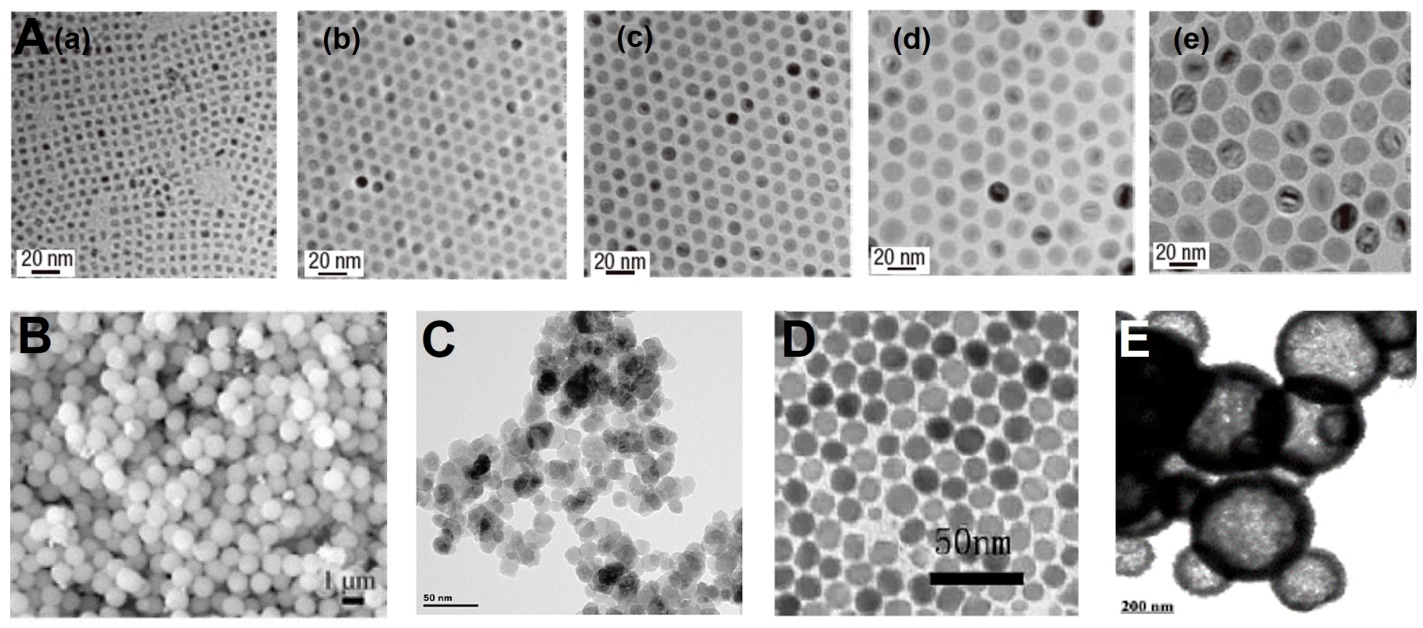
(A) TEM images of monodisperse Fe_3_O_4_ nanocrystals synthesized by the thermal decomposition in solvent with an increasing boiling point: (a) 5 nm, 1-hexadecene (b.p. 274 °C); (b) 9 nm, octyl ether (b.p. 287 °C); (c) 12 nm, 1-octadecene (b.p. 314 °C); (d) 16 nm, 1-eicosene (b.p. 330 °C) and (e) 22 nm, trioctylamine (b.p. 365 °C). Adapted with permission from 88, copyright 2004 Nature Materials. (B) SEM images of Fe_3_O_4_ NPs synthesized by solvothermal reaction. Adapted with permission from 90, copyright 2005 Angewandte Chemie. (C) TEM images of Fe_3_O_4_ NPs synthesized by hydrothermal reaction. Adapted with permission from 92, copyright 2013 Biomaterials. (D) TEM images of Fe_3_O_4_ NPs synthesized by microemulsion synthesis. Adapted with permission from 95, copyright 2006 Advanced Functional Materials. (E) TEM images of mesoporous Fe_3_O_4_ NPs synthesis by hard templates. Adapted with permission from 97, copyright 2008 Advanced Materials.

Thermal decomposition of organometallic precursors into metal oxides or metal elements at high temperature is a classic method to synthesize monodisperse nanocrystals 79, 88, 89. Due to the presence of organic ligands, the aggregation and overgrowth of nanoparticles are restricted and the diameter of nanoparticles is closely related to the reaction time and the boiling point of organic solvent 98. Generally, the metal complex precursors are rapidly injected into hot organic solvents or directly heated up with the solvents 99, 100. In the hot injection process, after the metal precursors solution is rapidly injected into the solvents at the reaction temperature, an instant nucleation immediately happens with a controlled growth process. In the heating-up procedure, the nucleation increases gradually with the raise of the reaction temperatures 101-103. Oleic acid (OA), oleylamine (OAm) and 1-octadecene (ODE) are the commonly used solvents as well as surfactants to stabilize nanoparticles and control their sizes and morphologies 104. It is worth mentioning that the surface of nanoparticles prepared by this method is hydrophobic, and subsequent surface modification is needed to improve the hydrophilicity for the utilization in biological environment 105. Hyeon *et al*. first prepared uniform and small-sized monodisperse Fe_3_O_4_ nanocrystals (4-12 nm) through this method 88, 89. The size of the Fe_3_O_4_ nanocrystals were controlled by the boiling points (b.p.) of the organic solvent. As shown in Figure 2A, the size of Fe_3_O_4_ nanocrystals increases with the rise of solvent boiling point. This method has been widely used for synthesis uniform small-size Fe_3_O_4_ NPs with good crystallinity and be widely used in the application of MRI contrast agent, drug carrier, further assembly forms cluster structure use for photothermal or magnetic hyperthermia treatment of tumors, which shows great significance to the biological application of Fe_3_O_4_.

### Solvothermal reaction/Polyol method

In order to prevent the self-aggregation and over-growth of monodisperse Fe_3_O_4_ nanocrystalline during the synthesis process, the decomposition of metal salts in organic solution-phase have been widely used 88, 89. Based on this principle, the solvothermal method is extensively used to synthesize Fe_3_O_4_ NPs with low cost, simple operation, and excellent crystallinity. Li *et al.* 90 reported the method for the synthesis of hydrophilic, monodisperse, and single-crystalline magnetic ferrite (MFe_2_O_4_; M=Fe, Mn, Zn, or Co) microspheres by a solvothermal reduction method, and the monodispersed ferrite spheres were controlled with the diameters from 200 to 800 nm (Figure 2B). This solvothermal method provides an important method to get monodisperse nanostructures without demanding a narrow size distribution. However, the final nanocrystals from oleic acid/amine during the solvothermal reaction usually coated with long alkyl chain oleic acids or amines with hydrophobic surface properties, which greatly inhibits their applications in biotechnological areas and others. In order to receive monodisperse and hydrophilic nanocrystals, ethylene glycol could be used as the solvent in the solvothermal synthesis of magnetic ferrite nanocrystals with hexanediamine or polyethylene glycol as protecting reagents.

The polyol method is similarly developed to fabricated Fe_3_O_4_ NPs by using polyols (such as, diethylene glycol, ethylene glycol and triethylene glycol) to reduce the metal compounds to obtain the corresponding nanoparticles. In this method, the solvent of polyols with high boiling points can be used as both the reducing agents and the stabilizing agents to control the growth process of nanoparticles and prevent possible agglomeration 91. Wan *et al.* 106 prepared monodisperse Fe_3_O_4_ NPs with a uniform size of 10 nm through the polyol method using the triglyceride glycol and iron acetylacetone, the obtained Fe_3_O_4_ NPs showed well dispersity in aqueous or other polar media due to hydrophilic polyol ligands coated in the synthesis process.

### Hydrothermal reaction

Fe_3_O_4_ NPs can also be synthesized by the hydrothermal reaction under the condition of suitable temperature (100-250 ℃) and relatively high pressure (0.3-4 MPa) 107. Hydrothermal synthesis involves no organic solvents or metal organic precursors, and the high crystallinity and good hydrophilicity of yielded Fe_3_O_4_ NPs omits the subsequent surface modification processes 92. Shi *et al.* 92 reports the facile preparation of Fe_3_O_4_ NPs coated with branched PEI (abbreviated as: Fe_3_O_4_-PEI NPs)* via* the hydrothermal method (Figure 2C). The surface of Fe_3_O_4_-PEI NPs with primary amine groups, which could be further modified with PEG, succinic anhydride and acetic anhydride, and induce Fe_3_O_4_-PEI NPs continue surface functionalities. Hydrothermal reaction provides a choice for the simple synthesis Fe_3_O_4_-based multifunctional nanomaterial for biomedical applications.

### Coprecipitation synthesis/Sol-gel synthesis

Coprecipitation of Fe^2+^ and Fe^3+^ ions in solution also a classical method applied to manufacture Fe_3_O_4_ NPs by precipitating a specific proportion of the inorganic salts in aqueous media 79, 108. Compared with the thermal decomposition synthesis, the coprecipitation can avoid the problem that surface decoration (template molecules, surface organic ligands, surfactant) are difficult to be removed 93, 94. The size, quantity and morphology of the obtained Fe_3_O_4_ NPs could be controlled by experimental condition, such as power of hydrogen (pH), ion concentration, reaction temperature, precursor and so on. Stroeve *et al.* 109 synthesized the Fe_3_O_4_ NPs in aqueous solutions without any surfactants. The coprecipitation process of Fe^2+^/Fe^3+^ was achieved by changing the pH value of solution, and the achieved Fe_3_O_4_ NPs with a narrow size distribution and the average diameter less than 10 nm 98.

Sol-gel method can be considered as the further development from this strategy, which stirred the metal salt with a gelling agent to form a homogeneous gel, then gelled the sol by chemical reaction or solvent removal to get a 3D iron oxide network 96. To obtain the pure Fe_3_O_4_ NPs, the formed gels usually requires an additional crushing step after drying and calcination. The structure and properties of the obtained Fe_3_O_4_ NPs are usually influenced by the concentration, reaction temperature, pH values, and solvents. The yield of nanoparticles using the sol-gel method usually is high, thus this method can be used for massively producing large-sized nanoparticles.

### Microemulsion synthesis

Microemulsion method is also widely investigated as a classic method to prepare nanocrystals 110. Microemulsion is a thermodynamically stable dispersions, which can be obtained by mixing immiscible water/oil phase that stabilized by the arrangement of co-surfactant or surfactant molecules at the interface. The microemulsion system especially the water-in-oil (W/O) phase, which is consist of the dispersion of water nanodroplets in the oil phase to form a stabilized spherical reverse micelle, can be considered as “nanoreactor” for the synthesis of nanoparticles. Since the nucleation and growth of nanoparticles are limited in the nanoreactor, that the size of nanoparticles can be controlled 111. Li *et al.* 95 reported the synthesis of the magnetic Fe_3_O_4_ NPs by the W/O microemulsions, and investigated the relationship between the experiment condition (concentration, water/ethanol/organic solvent ratio, kinds of surfactants, temperature, reaction time) and the properties (morphology, crystal phase, and size distribution) of the obtained Fe_3_O_4_ NPs (Figure 2D).

### Template synthesis

Recently, many researches have designed and prepared of hollow or porous Fe_3_O_4_ nanostructures by the template synthesis method, which can be divided into the hard template method (e.g., silica, carbon spheres, and polymer nanoparticles) and the soft template method (e.g., vesicles, micelles, emulsion droplets, gas bubbles and others) according with the process of package, calcination and separation in obtaining the hollow nanostructure 112. Among the hard templates, polymer latex particles, especially polystyrene (PS) beads, have been demonstrated to be effective templates for the preparation the hollow spherical inorganic materials of Fe_3_O_4_ NPs 113, and this method also be applied to other nanoparticles, such as Gd_2_O_3_ 105, TiO_2_ 114, ZnS 115, SiO_2_ 116 and so on. Similarly, mesoporous silica can also be used as the hard templates for the preparation of mesoporous Fe_3_O_4_ NPs, followed by the process of heating treatment and silica removal (Figure 2E) 97. Compared to the hard template method, the morphology of Fe_3_O_4_ NPs synthesized by the soft template method is usually more uncontrollable. The obtained Fe_3_O_4_ NPs usually present nanowires structure and are rarely used in cancer diagnosis and treatment.

## Surface Modification of Magnetic Fe_3_O_4_ NPs

Fe_3_O_4_ NPs prepared with hydrophobic ligands (oleic acid or stearic acid) needed to be converted into hydrophilic ligands for further biomedical applications 104. Meanwhile, appropriate surface modification can also import the Fe_3_O_4_ NPs with better biocompatibility, long blood circulation and further functionalization, such as active targeting ability 105, 117. Currently, the surface modification methods of Fe_3_O_4_ NPs fall into two categories of ligand replacement and encapsulation 118, 119. Ligand replacement refers to an exchange of native hydrophobic ligands (oleic acid) on the surface of nanoparticles with strong anchoring groups (such as phosphonates, catechols, thiols, sulfonates, and carboxylic acids), which act as hydrophilic ligands to improve their dispersity in biological environment 120-123. However, the actual ligand exchange process is often underperformed and always causes the leakage of surface ligand defectiveness. In contrast, the surface modification of Fe_3_O_4_ NPs through the encapsulation method shows better efficient, which can effectively coating the surface of Fe_3_O_4_ and ensure their uniformity 124, 125. The usual strategy of encapsulation process is to utilize inorganic or organic shells to embellish iron oxide nanoparticles into core-shell structured nanocarriers 126-128, and the surface functionalized decorations mainly include noble metals (e.g., gold, silver, gadolinium) 129-132 or oxides (e.g., silica, graphene, titanium dioxide) 133, 134, biodegradable organic polymers (e.g., polyethylene glycol (PEG), poly (lactic-coglycolicacid) (PLGA)) 135-137, proteins (e.g., antibodies, monoclonal antibody and their fragments) 138, nucleic acids (e.g.*,* DNA, siRNA, aptamers) 134, 139-141, amino acids (e.g.*,* phenylalanine, tyrosine, arginine, lysine and cysteine) 142, small molecules (e.g., photosensitizer, folic acid, doxorubicin), and other species (vitamins, carbohydrates) 143, 144. Here we detailly introduce the recent advances in the surface modification Fe_3_O_4_ NPs (Table 3).

### Inorganic mesoporous materials

**Figure 3:**
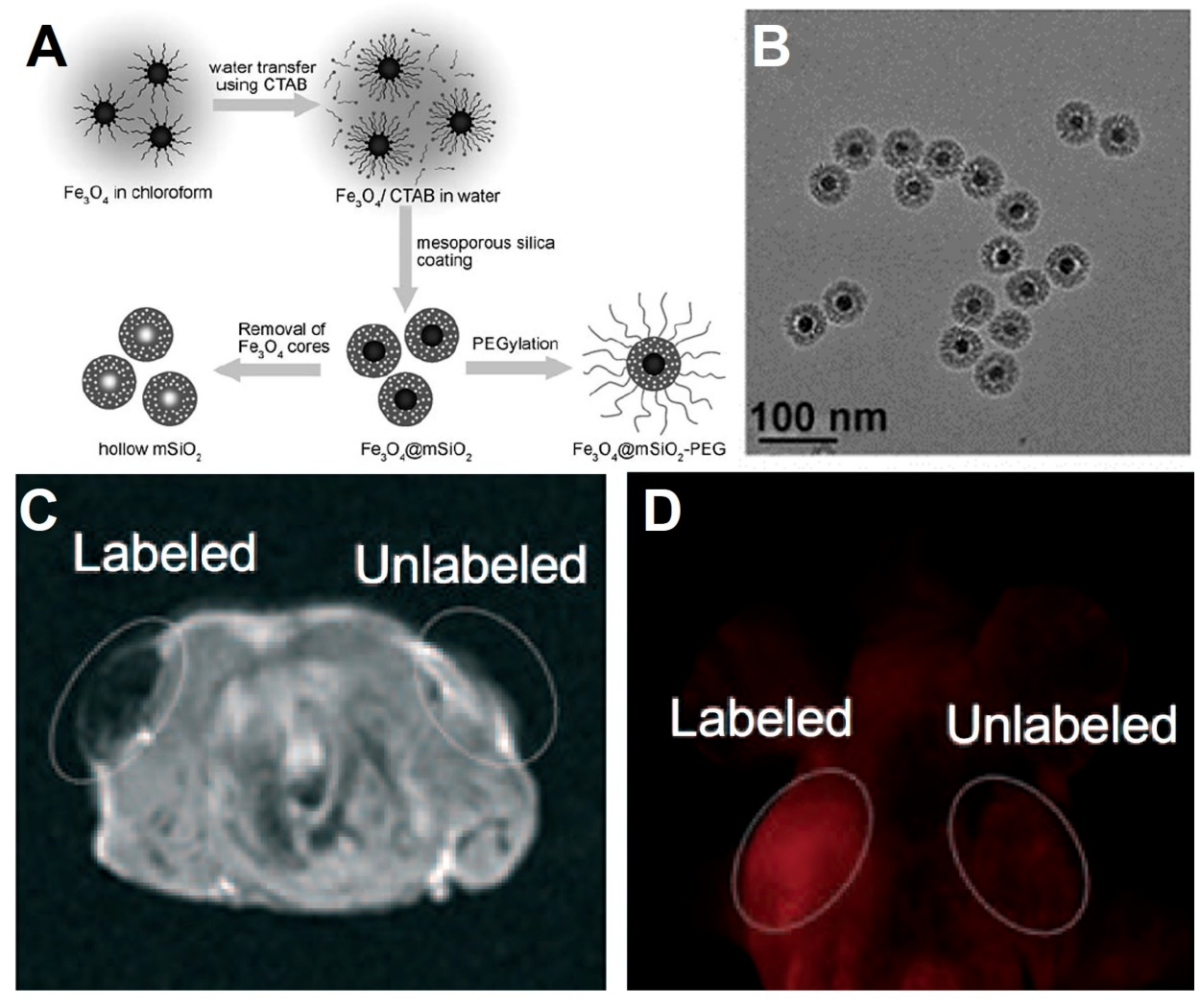
(A) Synthetic schematic illustration of core-shell structure Fe_3_O_4_@mSiO_2_ NPs. (B) TEM image of Fe_3_O_4_@mSiO_2_ NPs. *In vivo* imaging for the *T*_2_-weighted MRI imaging (C) and fluorescence confocal microscopy image (D) using Fe_3_O_4_@mSiO_2_. Adapted with permission from 153, copyright 2008 Angewandte Chemie International Edition.

In recent years, many inorganic nanomaterials have been extensively studied in biomedical fields, such as silica, aluminum oxide, molybdenum dioxide, graphene, calcium carbonate, calcium phosphate and others 145-147. Among them, mesoporous silica (mSiO_2_) materials have been used as delivery carriers for small molecule drugs, quantum dots, siRNA and aptamers due to their uniform pore sizes and high accessible pore volume 148, 149. Meanwhile, the monodisperse mSiO_2_ smaller than 100 nm, have also been proved to possess a high stability in blood circulation 150-152. The modification of mSiO_2_ onto Fe_3_O_4_ NPs to form uniform core-shell nanocomposite have been reported to improve their biocompatible, enhance hydrophilicity and provide anchoring points (Si-OH groups) for further loading of molecular drugs (e.g. paclitaxel, platinum-based drugs, DOX) or other targeted functional groups (e.g. FA, antibodies). The modification of mSiO_2_ shell onto Fe_3_O_4_ NPs usually using the sol-gel method or microemulsion method. Hyeon *et al.* 153 synthesized the composite structure consist of monodisperse Fe_3_O_4_ NPs core and mSiO_2_ shell (Figure 3). CTAB was used as the surfactant for the transfer of hydrophobic Fe_3_O_4_ NPs, and provide the soft template for the grown of mSiO_2_ shell in the sol-gel process. The size of Fe_3_O_4_@mSiO_2_ NPs could be controlled within 100 nm, and the fluorescein isothiocyanate and rhodamine B isothiocyanate were further modified on the mSiO_2_ shell for fluorescence imaging *in vivo,* which could show typical emissions of fluorescein and rhodamine B at 516 nm and 577nm with the corresponding excitation wavelength of 460 nm and 520 nm. Meanwhile, the core-shell Fe_3_O_4_@mSiO_2_ NPs also could be used as the contrast agent for *T*_2_-weighted MRI *in vivo*, providing an idea for construction multimodal imaging platform by modifying the hydrophobic Fe_3_O_4_ NPs with the functional mesoporous material shell.

### Noble metals

Magnetic Fe_3_O_4_ NPs, due to their excellent imaging properties and magnetic targeting ability, are usually conjunction with other functional systems to improve their capability for multimodal imaging and precision targeting 154. Among them, noble metals are a crucial species of functional components. On one hand, most of noble metals have a higher atomic number than the iodine element, and can be used as the contrast agent for computed tomography (CT) imaging, due to their remarkable X-ray attenuation property 155, 156. On the other hand, noble metals usually have the strong surface plasmon resonance (SPR) effect, which could absorb the energy of light and convert that into thermal or other energy 157. In particular, the core/shell nanostructures adopt the plasmonic effects of noble metals into magnetic Fe_3_O_4_ NPs, can be used in the photothermal therapy of cancer 158-160. Shi *et al.* 157 developed a multifunctional theranostic nanoplatforms for tumor imaging and therapy based on the star-shaped Fe_3_O_4_@Au core/shell nanoparticles, which presented an excellent effect in MRI, CT, thermal imaging and photothermal therapy. After further modification of PEI and HA, the nanostars showed better biocompatibility, stability and targeting for cancer cells.

**Figure 4:**
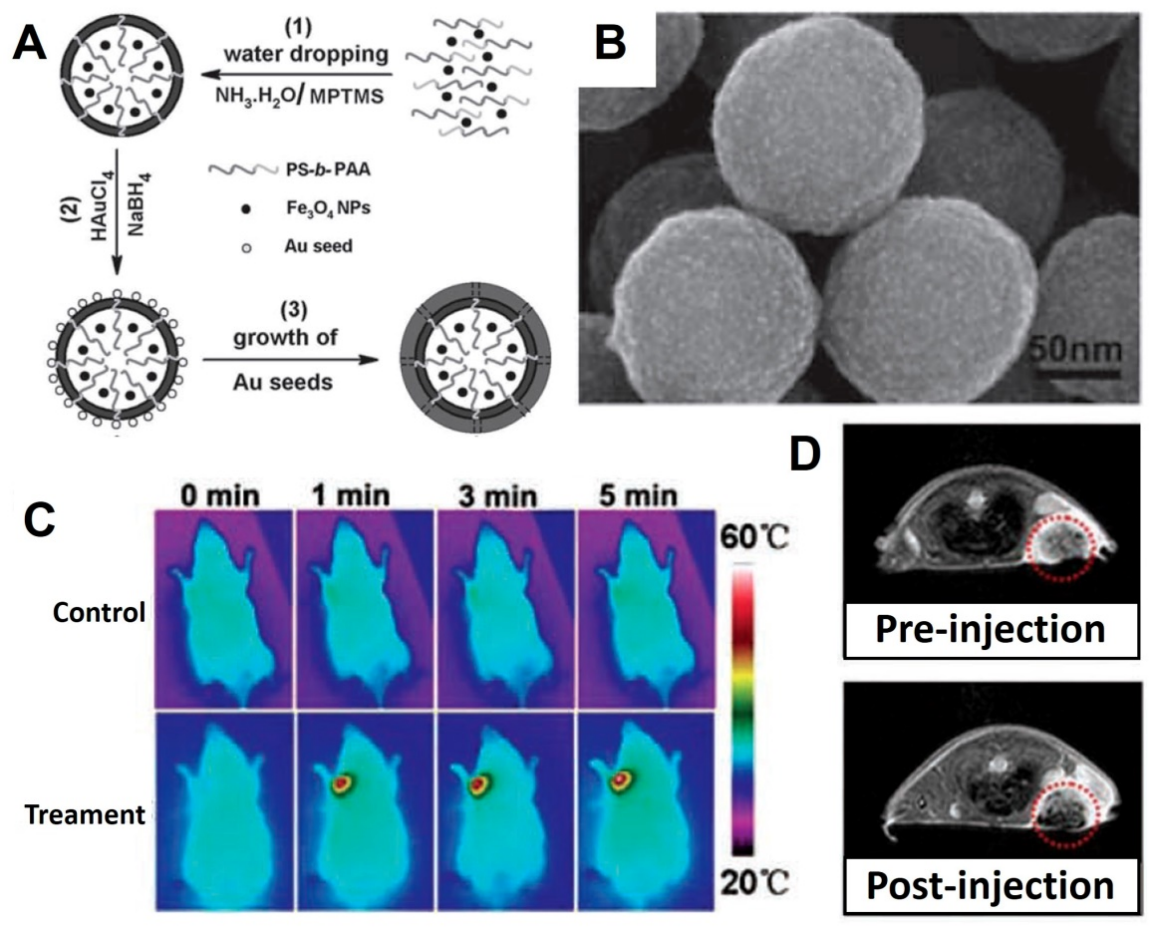
(A) Schematic of synthesis Fe_3_O_4_/Au nanocomposite. (B) SEM images of Fe_3_O_4_/Au core-shell structure. (C) Thermal images effect of Fe_3_O_4_/Au nanocomposite under 808 nm irradiation. (D) The *T*_2_-weighted MRI effect of Fe_3_O_4_/Au nanocomposite *in vivo*. Adapted with permission from 158, copyright 2011 Advanced Materials.

Shi *et al.* 158 reported a method for preparation the core-shell structured of Fe_3_O_4_/Au, and the Fe_3_O_4_ NPs core are linked to the Au shell by the hybrid of SiO_2_ and PS-b-PAA (a kind of amphiphilic block copolymers consisting of polystyrene and polyacrylicacid). The details of synthetic process are shown in Figure 4A. First process is modified SiO_2_ on the surface of Fe_3_O_4_ NPs and self-assembly with the PS-b-PAA. Then reduce AuCl_4_^-^ on the surface of Fe_3_O_4_@hybrid to Au NPs form the shell structure, and ultimate formation the Fe_3_O_4_/Au. The SPR effect of Au NPs on the core-shell structured could absorb the near-infrared light (808 nm) and converted that into thermal energy and eventually kill the tumor cells. Meanwhile, due to the MRI performance of the Fe_3_O_4_, the noble metal modified Fe_3_O_4_ not only have property of photothermal therapy, but also an agent of MRI and thermal imagery, demonstrating a flexible way for the construction of Fe_3_O_4_-based multifunctional diagnosis and treatment platforms.

### Polymers

**Figure 5:**
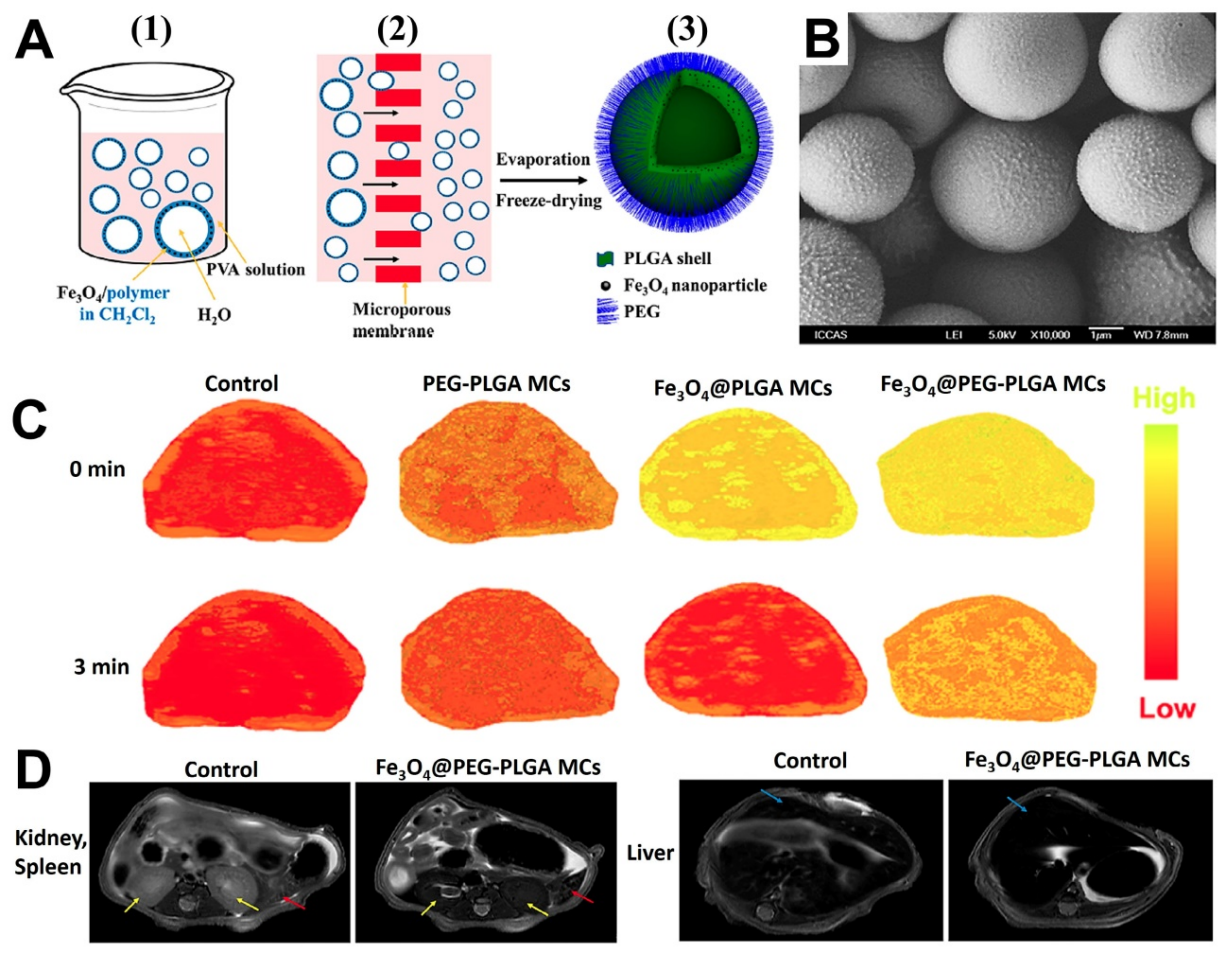
(A) The schematic diagram: (1) The traditional method yielded microcapsules with an uneven distribution; (2) Premix membrane emulsification method obtained uniform microcapsules with the help of microporous membrane; (3) The structure diagram of Fe_3_O_4_@PEG-PLGA MC. (B) SEM images of Fe_3_O_4_@PEG-PLGA MCs. The imaging of *in vivo* USI (C) (liver) and MRI (D) of mice. Arrows of yellow, red and blue corresponding to kidneys, spleens and livers. Adapted with permission from 170, copyright 2015 ACS Applied Materials & Interfaces.

In the last decade, biocompatible polymers have been extensively used to improve conventional mode of medication in drug-delivery 161-163. Meanwhile, the magnetic Fe_3_O_4_ NPs are gaining more research interests in the medical fields relying on their unique properties such as imaging capability of MRI and magnetic targeting drug carrier. For improving the hydrophilicity and other physiochemical properties of Fe_3_O_4_ NPs, the surface modification with polymers provided an ideal choice, and the commonly used polymers include polyethylene glycol (PEG), poly(L-lysine) (PLL) 164, poly(propyleneimine) (PPI), polyethyleneimine (PEI), polystyrene (PS) 165, poly (vinyl pyrrolidone) (PVP), poly (lactic-co-glycolic acid) (PLGA) and poly (vinyl alcohol) (PVA)), which having hydrophilic segments to improve biodegradability and biocompatibility. Parveen *et al.* 166 revealed the surface coated Fe_3_O_4_ NPs with the hydrophilic PEG could prevent nanoparticles from being removed by the phagocytic activities to further boost the endurance of the Fe_3_O_4_. PLGA is a biodegradable polymer and has been frequently used as a drug-delivery platform in the treatment of cancer therapy in recent years, and has also been reported to modify magnetic Fe_3_O_4_ NPs for enhance their magnetic targeting delivery ability to tumor tissues under magnetic field conditions 167-169. Wu *et al.* 170 prepared the uniform microcapsules with Fe_3_O_4_ and PEGylated PLGA (abbreviated as Fe_3_O_4_@PEG-PLGA MCs) for MRI and ultrasound bimodal imaging (USI) (Figure 5). The Fe_3_O_4_@PEG-PLGA MCs showed better stability in physiological solutions owing to the PEGylation. Meanwhile, the *in vivo* and *in vitro* experiment results showed the polymers modified Fe_3_O_4_-based microcapsules could be used as an agent for USI and MRI performance, without dramatic cytotoxicity and embolism to mice even at high doses.

### Metal-organic frameworks

**Figure 6:**
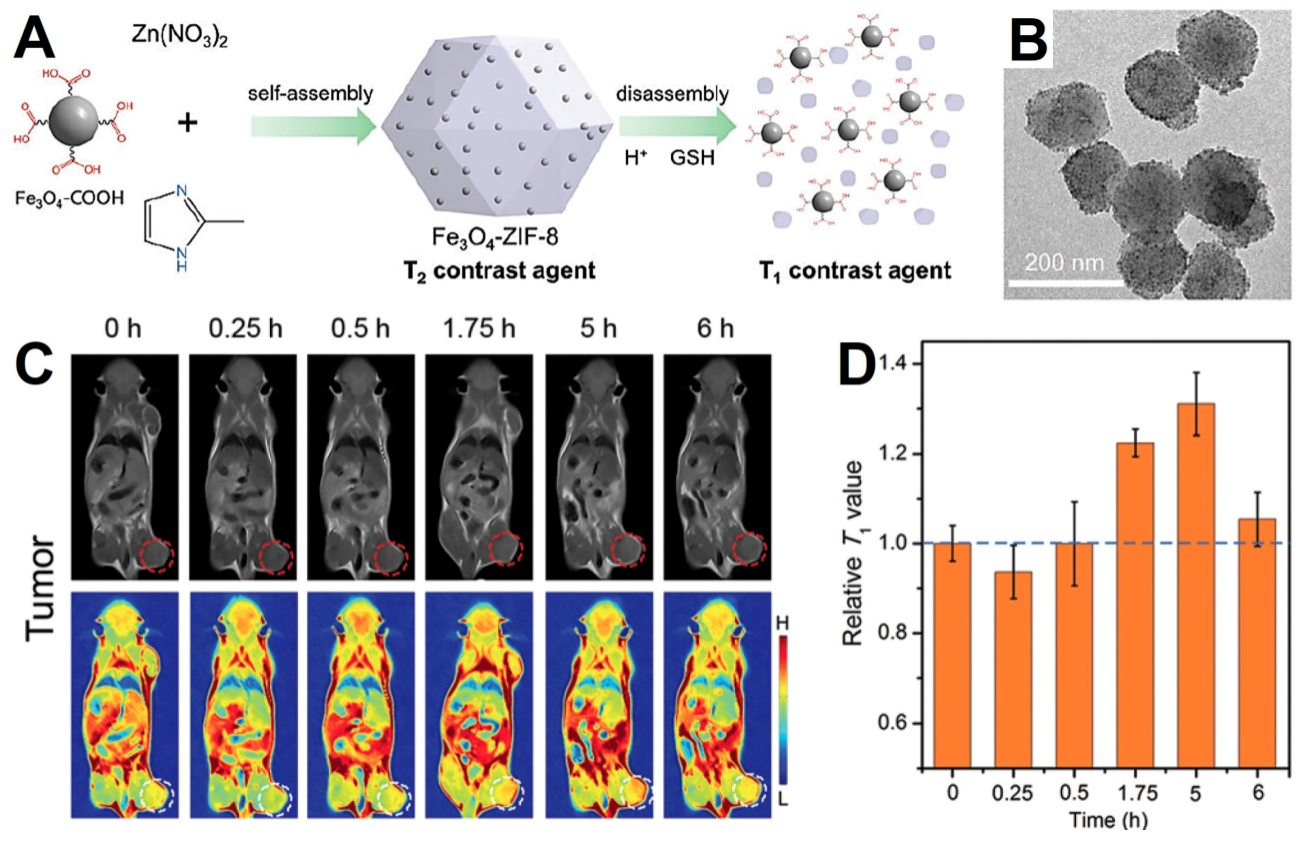
(A) Schematic of Fe_3_O_4_-ZIF-8 switch from *T*_2_ to *T*_1_ weight. (B) TEM images of Fe_3_O_4_-ZIF-8 assemblies. (C) *In vivo T*_1_-weight MRI of tumor after intravenous injection of Fe_3_O_4_-ZIF-8, and homologous *T*_1_ signals (D) extracted from tumor sites. Adapted with permission from 172, copyright 2019 Chemical Communications.

Metal-organic frameworks (MOFs) is an emerging species of porous nanomaterials acquire from metal ions or clusters coordinating with bridged ligands, which could be used to load guest nanoparticles to gain new performance due to their good physicochemical stability, larger surface area and tunable functionality 171. Responsive MOFs coated Fe_3_O_4_ NPs have been studied for improving the hydrophilicity, increasing the porosity of delivery system and enhancing the responsiveness of the tumor environment. The mechanism of MOFs responsive decomposition may be due to the effects of H^+^ and glutathione (GSH) in the tumor microenvironment on the binding of metal ions and organic ligands 171, 172. Yang *et al.* 172 designed a novel MRI contrast agent, which utilized the pH and GSH responsive ZIF-8 as nanocarrier to deliver small-sized Fe_3_O_4_ NPs (about 5 nm, *T*_1_-weight MRI) into a Fe_3_O_4_-ZIF-8 nanostructure (*T*_2_-weight MRI). The slightly acidic conditions and overexpressed GSH in tumor microenvironment would lead the decomposition of the Fe_3_O_4_-ZIF-8 nanostructure and release the small-sized Fe_3_O_4_ (Figure 6A), leading to the MRI effect from *T*_2_ to *T*_1_ and enhancing the contrast of the tumor tissue (detail in part 4.3), which could distinguish tumor tissue from the normal tissue by the *T*_2_ to *T*_1_ conversion. Han *et al.* 173 synthesized a novel Co-ferrocene metal-organic framework (Co-Fc MOF) with high Fenton activity. After combined with the glucose oxidase (GOx), the nanoplatform (Co-Fc@GOx) construct an enzymatic/Fenton catalytic synergistic effect for enhanced tumor treatment effect. GOx delivered by Co-Fc MOF could catalyze endogenous glucose of tumor microenvironment to H_2_O_2_ and gluconic acid, which further favored the Fenton reaction of Co-Fc MOF and enhanced the generation of ROS. Experimental results demonstrated this synergistic enzymatic/Fenton catalytic activity triggered by Co-Fc@GOx nanoplatform enabled remarkable anticancer properties both* in vivo* and *in vitro.*

### Cell membranes and derivatives

**Figure 7:**
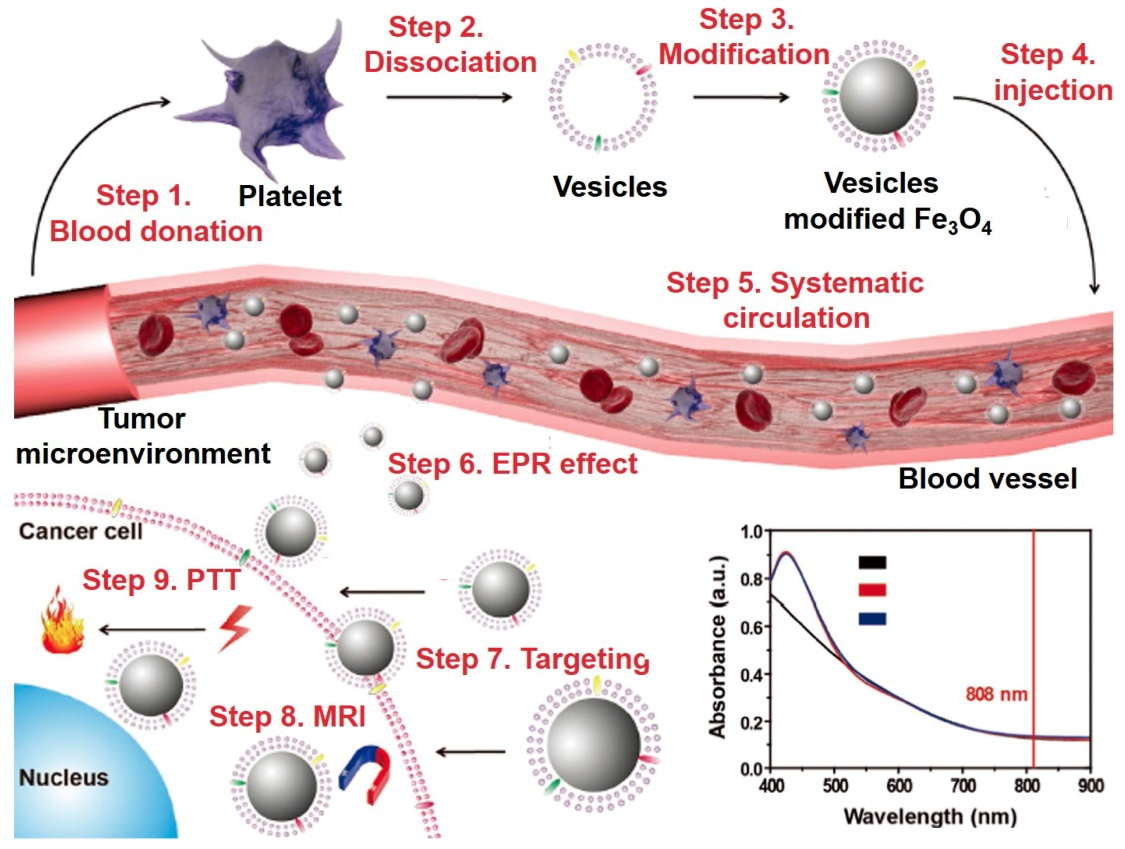
The schematic of platelet vesicles modified Fe_3_O_4_ NPs for enhanced the effect of MRI and PTT, and the UV-Vis-NIR absorption spectra of Fe_3_O_4_ NPs. Adapted with permission from 183, copyright 2017 Advanced Functional Materials.

It is a common method to functionalize the nanoparticles with tumor imaging and therapy with the exogenous ligand. However, several problems are still needed to be solved, such as: (1) the foreign nanoparticles can be easily detect by immune systems and cause severe immune responses; (2) the physiological barriers could eliminate nanomaterials from the blood circulation and restrict the accumulation in target sites; (3) nontargeted nanomaterials that relies on the EPR effect limit the therapeutic effects and increase the damage to normal tissues; (4) the potential toxicity of nanomaterials* in vivo* 174-180. Fe_3_O_4_ NPs with their unique properties and long blood circulation time are widely researched to overcome these limitations. Compared to exogenous ligands for surface modification, the endogenous cell membranes for nanoparticles wrapping provide a novel method for solve these problems 181-184. The classic types of biomimetic cell membranes include red blood cell membrane, white blood cell membrane, cancer cell membrane, stem cell membrane and so on 185. Zhao* et al.* 183 fabricated the delivery platform based on the platelet mimicking Fe_3_O_4_ NPs for enhancing the blood circulation time and targeting ability. As shown in Figure 7, step 1 showed the collections of blood from the mice, step 2 showed the dissociation of membrane and protein from platelet formed the vesicles, step 3 was to coat the vesicles of platelet on the surface of Fe_3_O_4_ NPs, step 4 was to injection the vesicles modified Fe_3_O_4_ NPs into blood vessel, steps 5 to 7 were the systematic circulation of vesicles modified Fe_3_O_4_ NPs, enrichment in tumor tissues by EPR effect and entry into tumor cells with the help of vesicles, steps 8 and 9 showed the performance test of imaging and therapeutic by the MRI and photothermal therapy (PTT). The results showed that the cell membrane modified Fe_3_O_4_ NPs have a good tumor targeting ability and could kill the tumor cells by photothermal treatment under the irradiation of near infrared light.

### Aptamers

In general, when the Fe_3_O_4_ NPs circulate in the blood system, they are easily subjected to macrophage uptake and reticuloendothelial system clearance. To improve specificity and recognition of the tumor targeted delivery system, many functionalized substances such as aptamers, peptides and small molecule can also be used for the surface modification of nanoparticles. Aptamer is a single-stranded oligonucleotide generated from an *in vitro* selection process, which called systematic evolution of ligands by exponential enrichment (SELEX) and they could bind with the targeted small molecules, proteins, and even intact cells and tissues with excellent specificity and high affinity. Aptamers used for surface modification have many advantages, such as easy to operate, good stability, rapid tissue penetration and lack of immunogenicity, thus making them as a suitable candidate. Tan *et al.* 186 designed a multifunction delivery platform that perform five distinct functions synergistically and effectively. To accomplish this, they prepared the gold-coated rose-shaped Fe_3_O_4_ (Au@Fe_3_O_4_) nanoplatform as an agent for photothermal therapy (PTT) and MRI. To enhance the targeting ability, the sgc8 aptamers were easily conjugated with the Au@Fe_3_O_4_ *via* the thiolate bonding, which could specifically recognize CCRF-CEM leukemia cells. Additionally, the chemotherapeutic agent doxorubicin (DOX) could also intercalate into the GC base pairs in the extended part of the aptamers, and with the releasing of DOX, the fluorescence signal *in vitro* also changed and cause the optical imaging. Therefore, the DOX-loaded Au@Fe_3_O_4_ nanoplatform presented five distinct functions for simultaneous imaging and therapy with the help of aptamers.

## Fe_3_O_4_ NPs for Tumor Imaging

### *T*_2_-weighted magnetic resonance imaging

MRI is an effective method for tumor diagnosis due to the acquired high contrast images and the precise handling of details of the targeted tissues with non-invasiveness and real-time monitoring 184. The sensitivity of MRI could be significantly enhanced with the help of contrast agents. The agents of MRI could be classified base on the effect on longitudinal (*T*_1_) or transversal (*T*_2_) relaxations, and the ability is defined as relaxivity (*r*_1_, *r*_2_). Therefore, MRI contrast agents could be divided in two types, which are *T*_1_-weighted (positive) and *T*_2_-weighted (negative), mainly shortens the *T*_1_ and *T*_2_ contrast media. In general, fast *T*_1_-weighted results appear to be bright contrast in the MRI, while the opposite *T*_2_-weighted results to be the dark contrast 187. Due to the superparamagnetic property, Fe_3_O_4_ NPs typically decrease the relaxation time of surrounding protons, thus providing possibility to be employed as *T*_2_-weighted MRI agent media.

**Figure 8:**
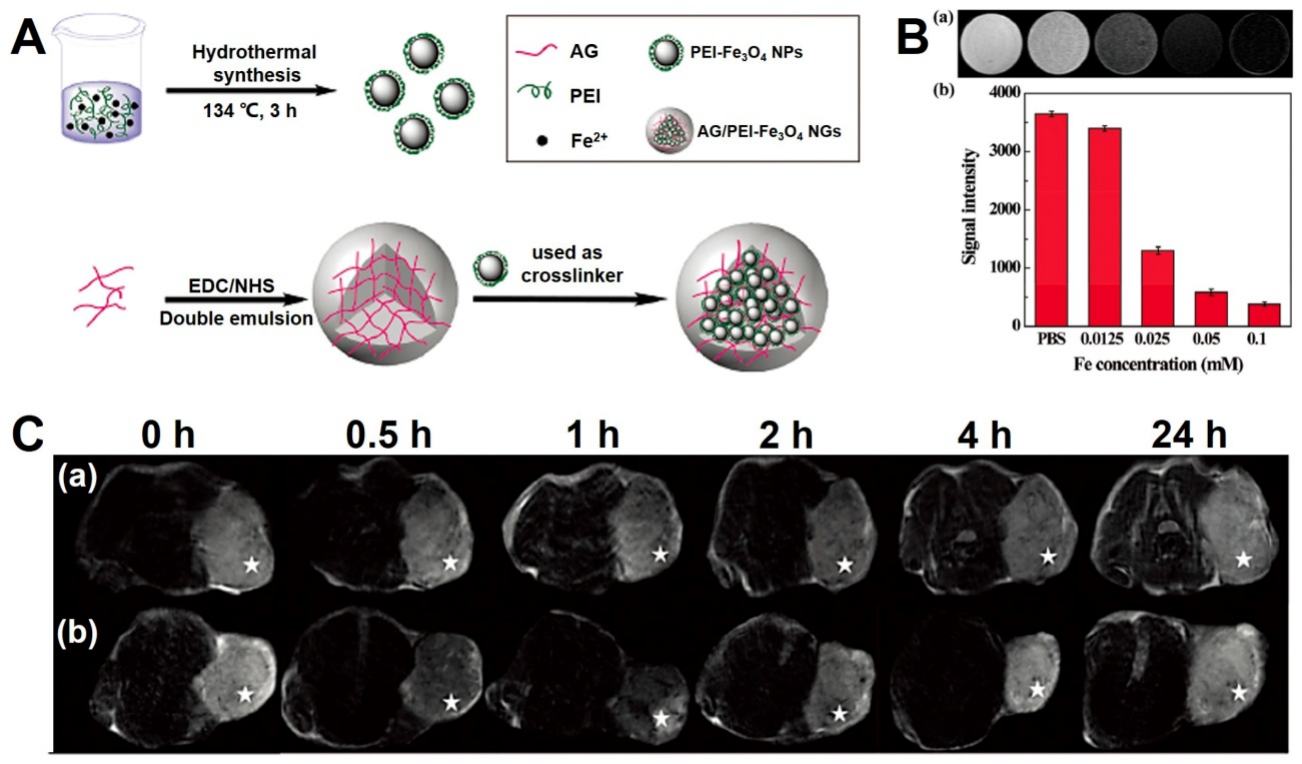
(A) The schematic of the synthetic process of AG/PEI-Fe_3_O_4_ NGs. (B) The imaging of *T*_2_-weighted MRI* in vitro* (a) and signal intensity analysis (b). (C) The images of *T*_2_-weighted MRI *in vivo*. Adapted with permission from 188, copyright 2016 Biomaterials Science.

Shi *et al.* 188 prepared a novel agent for MRI, which was the nanogels consisting of PEI coated Fe_3_O_4_ NPs immobilized with alginat (abbreviated as: AG/PEI-Fe_3_O_4_ NGs). The synthetic process is shown in Figure 8, and the nanogels presented a dispersion state in water with the size distribution from 153 nm to 219.2 nm. Moreover, the novel Fe_3_O_4_-based nanogels were excellent *T*_2_-weighted contrast agent for the MRI (the relaxivity of *r*_2_ is 170.87 mM^-1^s^-1^) and could be vastly swallowed by the tumor cells.

### *T*_1_-weighted magnetic resonance imaging

Superparamagnetic Fe_3_O_4_ NPs (SPIONs) are widely used as *T*_2_ contrast agents, since the strong magnetic moment could lead the magnetic inhomogeneity. However, some reasons limit their clinical application of *T*_2_ contrast agents 189. Because the intrinsic dark signal of *T*_2_-weighted MRI could not accurately distinguish the tumors and other hypointense areas, such as calcification, metal deposition, or bleeding. Meanwhile, the *T*_2_-weighted contrast agents usually with the high magnetic moment that perturbation the local magnetic field, which could exaggerate the size of the labeled area and blurs the images, thus causing the so-called “blooming effect”. Therefore, *T*_1_ contrast agents shows better desirable than *T*_2_ contrast agents for the accurate and high-resolution imaging 190. It is noteworthy that compared with the familiar *T*_1_ contrast agents, Fe_3_O_4_ contrast agents show better biocompatibility due to the iron element are rich in human blood, and stored in the body in the form of ferritin 201. However, the common Fe_3_O_4_ NPs are unfit for the *T*_1_ contrast agents because of that the ideal *T*_1_ contrast agents would have high *r*_1_ value and low *r*_2_/*r*_1_ ratio to realize the *T*_1_ contrast maximize effect 191. Although iron with five unpaired electrons which could increase the *r*_1_ value, the innate high magnetic moment of Fe_3_O_4_ NPs lead to the high *r*_2_ value and prevents then to be used as *T*_1_ contrast agent. However, this problem could be solved by decreasing the size of the Fe_3_O_4_ NPs, because the magnetic moment of Fe_3_O_4_ NPs could rapid decreases with the sizes reduction, which can lead to the reduction in the volume magnetic anisotropy 41, 192-194. Therefore, a series of research on Fe_3_O_4_ NPs as contrast agent are widely carried out.

**Figure 9:**
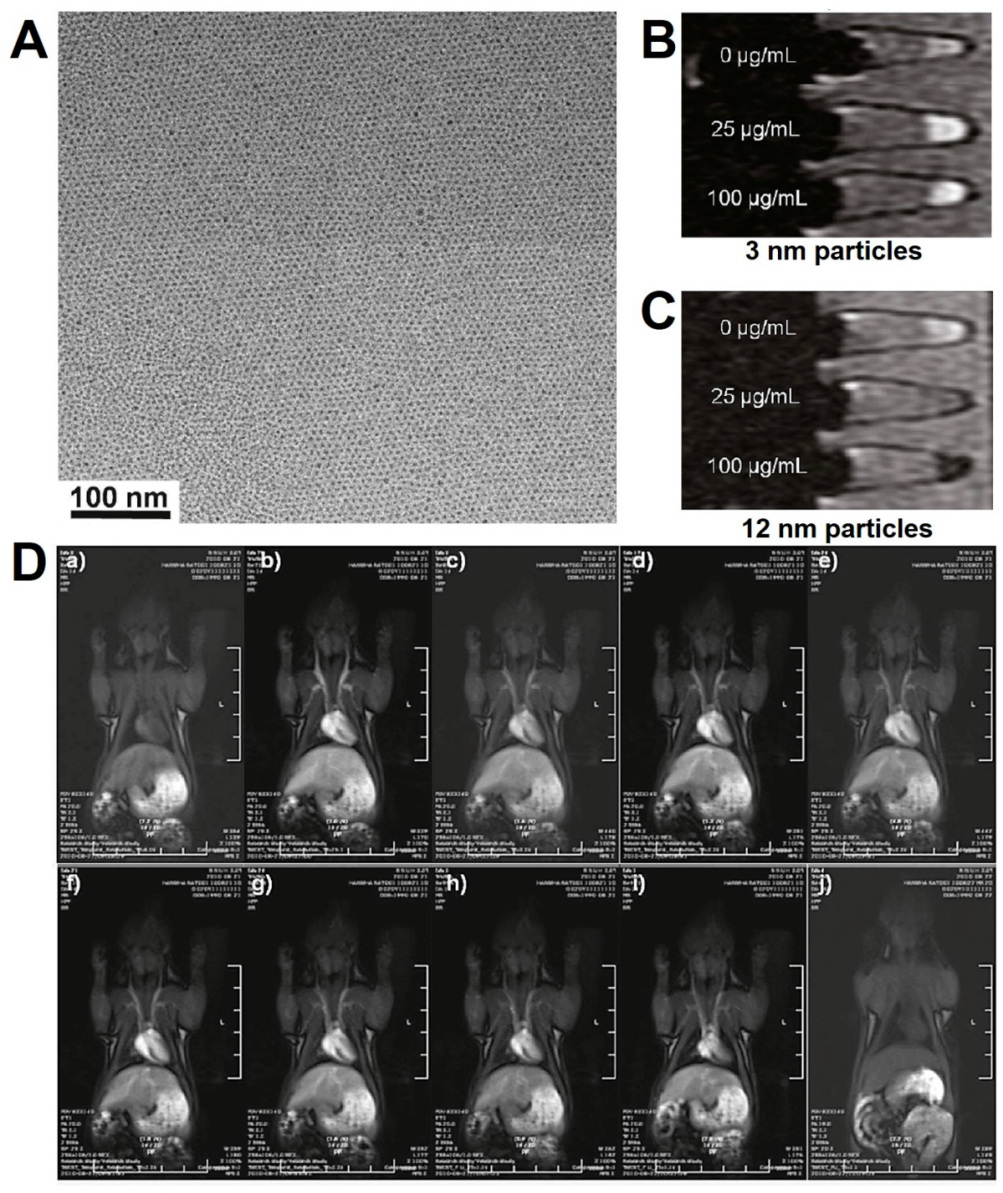
(A) TEM images of Fe_3_O_4_ NPs; *T*_1_-weighted MRI of Fe_3_O_4_ NPs with the diameters of 3 nm (B) and 12 nm (C); (D) The MRI intensity with the dynamic time *in vivo.* Adapted with permission from 189, copyright 2011 Journal of the American Chemical Society.

Hyeon *et al.* 189 reported the preparation of small-sized Fe_3_O_4_ NPs (mainly 3 nm) by the thermal decomposition (Figure 9), and the obtained Fe_3_O_4_ NPs showed extremely low magnetization derived due to the spin canting effect. For improving the water-dispersion and biocompatibility of Fe_3_O_4_ NPs, the PO-PEG ligands were modified on the surface. *In vitro* cytotoxicity assay of PO-PEG capped Fe_3_O_4_ NPs showed no observed toxic response, exhibited a high *r*_1_ relaxivity (4.78 mM^-1^·s^-1^) and low *r*_2_/*r*_1_ ratio (6.12), which demonstrated the magnetic Fe_3_O_4_ NPs could be used as *T*_1_ contrast agents. The high *r*_1_ relaxivity of Fe_3_O_4_ NPs could be attributed to the large surface number of iron with five unpaired valence electrons. The Fe_3_O_4_ NPs (around 3 nm) indicated a longer circulation time than the clinically used Gd-based contrast agents. High-resolution blood pool MRI using Fe_3_O_4_ NPs were able to provide a clear observation of various blood vessels within sizes less to 0.2 mm. All of these results demonstrated the Fe_3_O_4_ NPs with the potential as *T*_1_ contrast agents for MRI in clinic.

**Figure 10:**
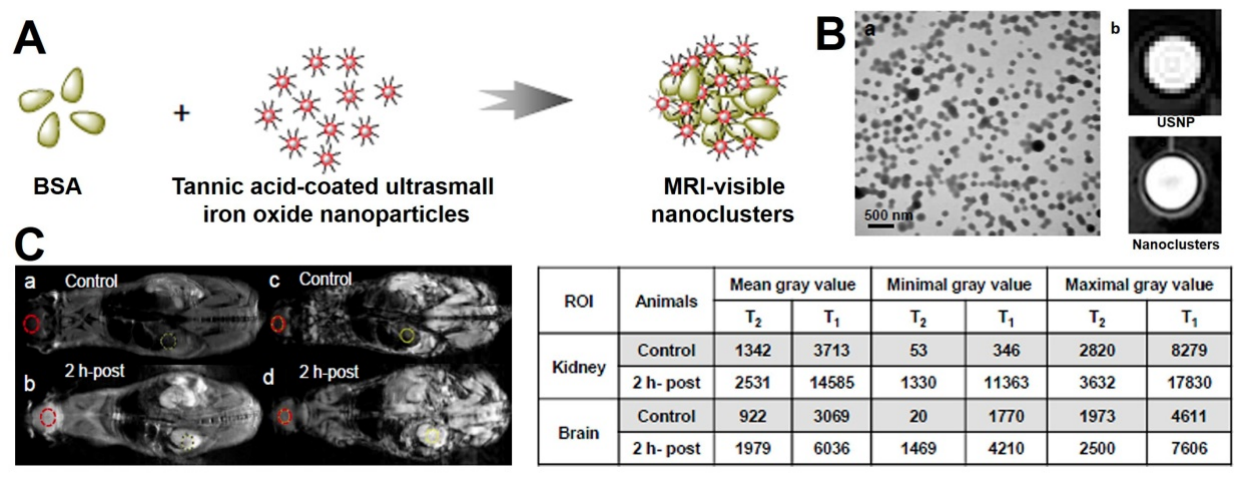
(A) The synthetic process of BSA modified Fe_3_O_4_ nanoclusters. (B) (a) TEM image of BSA modified Fe_3_O_4_ nanoclusters, (b) *T*_1_-weighted MRI of ultrasmall size Fe_3_O_4_ NPs (USNP) and BSA modified nanoclusters. (C)* T*_2_ and *T*_1_-weighted MRI after injection for 2 hours, the measured gray values of kidney and brain regions are shown in table. Adapted with permission from 196, copyright 2017 Nanoscale.

However, there are still several problems limit the ultrasmall size Fe_3_O_4_ NPs used as *T*_1_ contrast agents 195. First, ultrasmall size nanoparticles could be fast cleared out by renal metabolism and limit the imaging. Second, due to the high surface energy, the self-aggregation is a major concern of ultrasmall nanoparticles. Once aggregated the small Fe_3_O_4_ NPs will lose their *T*_1_ performance. Third, the surface modification of the ultrasmall size Fe_3_O_4_ NPs is critical to maintain the *T*_1_ performance, because the molecules modified on the surface directly controls the paramagnetic 195, 196. Therefore, it is necessary to modify the ultrasmall size Fe_3_O_4_ NPs reasonably while ensuring its performance. Bao *et al.* 196 reported a novel method to form nanoclusters by crosslinking bovine serum albumin (BSA) onto ultrasmall size Fe_3_O_4_ NPs (Figure 10). Different from traditional studies showing *T*_1_ signal decrease or complete loss after polymer encapsulation, the nanoclusters not only maintain the *T*_1_ contrast agent's performance of the Fe_3_O_4_ NPs, but also significantly enhanced the blood circulation times from 15 minutes to over two hours.

### 4.3 Responsive *T*_1_/*T*_2_ imaging

Due to the fact that magnetic Fe_3_O_4_ NPs display a remarkable change from the *T*_2_ enhanced contrast effect to *T*_1_ enhanced contrast effect when the size less than 5 nm, Fe_3_O_4_ NPs have become an attractive material for the preparation of responsive MRI contrast agents for tumor diagnosis. Yang *et al.* 172 utilized the ZIF-8 as a carrier for agglomerate small Fe_3_O_4_ NPs (a *T*_1_ contrast agent) into Fe_3_O_4_-ZIF-8 assembly (a *T*_2_ contrast agent). Because ZIF-8 is more stable under the normal physiological conditions and decompose under the acidic microenvironment of tumor tissues or in the presence of competitive ligands (Figure 6A). Therefore, the acidic conditions and GSH of the tumor microenvironment would trigger the disassembly of Fe_3_O_4_-ZIF-8 and to release the ultrafine size Fe_3_O_4_ NPs, leading the conversion from *T*_2_ to *T*_1_ enhancement on the sign of the tumor tissue.

**Figure 11:**
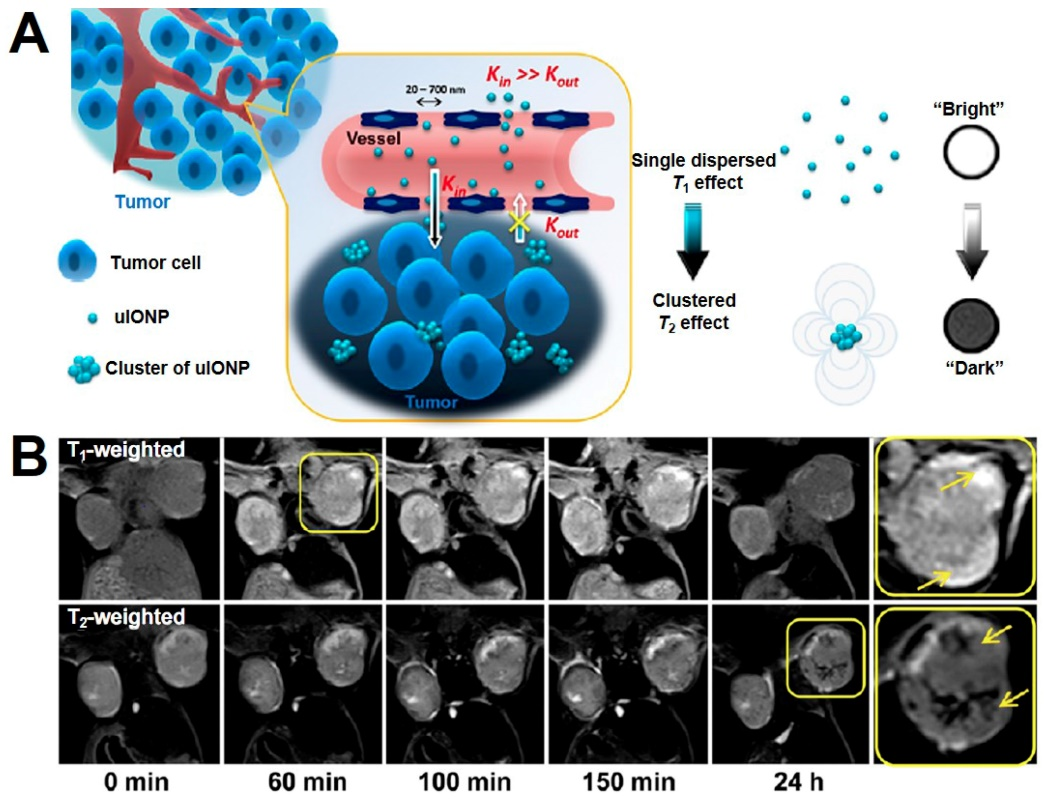
(A) The mechanism diagram of ultrafine Fe_3_O_4_ NPs (uIONPs) switch from *T*_1_-*T*_2_. (B) The change of *T*_1_ to *T*_2_-weighted MRI *in vivo* after injecting uIONPs for different times. Adapted with permission from 197, copyright 2017 ACS Nano.

The low delivery efficiency of nanomaterials is still a challenge in tumor imaging and treatment. Generally speaking, the EPR effect is considered as a driving force through either active or passive targeting for nanoparticles to reach and accumulate in the tumor tissues. Previous research has shown that nanoparticles in appropriate sizes (from 50 nm to 200 nm) can enhance the EPR effect through limit nanoparticle intravasate back into the circulation. Recently, more and more researches have been proved the mutual effect between nano system and the biological environment also affect the effect of nanomaterials and the mount of accumulation in tumor, some modified ultrafine size nanoparticles show better permeation and distribution in the tumor tissue than large nanoparticles, which include the size less than 5 nm Fe_3_O_4_* T*_1_-contrast agent. Mao *et al.* 197 reported a research using the Fe_3_O_4_ NPs (3.5 nm) modified with oligosaccharide (uIONPs), which could penetrate the tumor tissue and self-assemble in the acidic microenvironment of tumor tissues (Figure 11). The improvement on the delivery and tumor retention of Fe_3_O_4_ NPs were achieved by combining the reduced intravasation and enhanced extravasation. Moreover,* in vivo* MRI revealed that ultrafine Fe_3_O_4_ NPs showed “bright” *T*_1_ contrast when injected into the tumor vasculature, and then turn into “dark” *T*_2_ contrast after 24 h. The switch of *T*_1_-*T*_2_ contrast demonstrated the ultrafine Fe_3_O_4_ NPs with *T*_1_ contrast were dispersed state when inject into blood, and may aggregate formed large size Fe_3_O_4_ clusters with *T*_2_ contrast after penetrated into the tumor tissues. Therefore, this property of Fe_3_O_4_ NPs showed an inspiration on the design of responsive *T*_1_/*T*_2_ conversion MRI contrast agents.

### 4.4 Multimodal imaging

**Figure 12:**
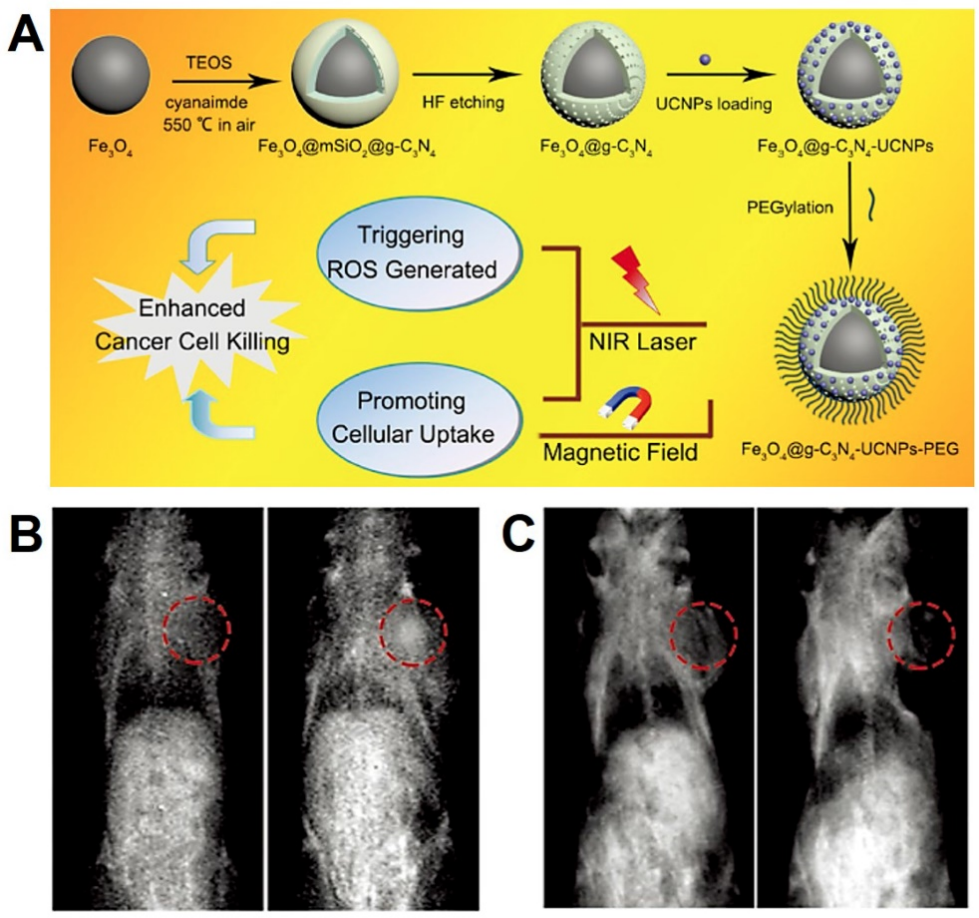
(A) The schematic diagram of synthesis process of Fe_3_O_4_@*g*-C_3_N_4_-UCNPs-PEG. (B.C) *In vivo T*_2_/*T*_1_-weighted MRI of samples, preinjected and after injection in situ. Adapted with permission from 200, copyright 2017 Advanced Healthcare Materials.

In the process of the diagnosis and treatment, single imaging modality often cannot provide complete information about the tissues and organs 194. Therefore, the combination of Fe_3_O_4_ NPs with two or more components to construct multimodal imaging system has become one of the main research directions 108. Liu *et al.* 198 have designed the core-shell structure of Fe_3_O_4_@Cu_2-x_S (<10 nm), which showed excellent superparamagnetic and photothermal conversion capability. Therefore, the Fe_3_O_4_@Cu_2-x_S nanoparticles could be used as the agent of MRI, thermal imaging and photothermal therapy. Lee *et al.* 199 designed a MRI agent by the construction of Fe_3_O_4_/MnO nanoparticles to implement the dual modes of *T*_2_ and *T*_1_ contrast enhancement of each compound. The *in vitro* and *in vivo* results that the dumbbell-shaped Fe_3_O_4_/MnO nanoparticles with negative *T*_2_ contrast effect in the full state, but in low pH environment, the positive effect of *T*_1_-weighted MRI was raised due to the releasing of Mn^2+^. Yang *et al.* 200 have designed a multifunctional nanoplatform by assembly the upconversion nanoparticles (UCNPs:* β-NaGdF_4_:Yb/Tm@β-NaGdF_4_*) on the surface of graphitic-phase carbon nitride (*g*-C_3_N_4_) coated Fe_3_O_4_ nanospheres (Figure 12). Due to the *T*_1_ contrast agents of Gd and *T*_2_ contrast agents of Fe, this platform showed the magnetic targeting ability under the guide of external magnetic field, and further supervised the therapeutic effect by dual-modal imaging precise localization.

## 5. Fe_3_O_4_ NPs for Imaging-Guided Tumor Treatment

### 5.1 Nanozyme

In recent years, Fe_3_O_4_ NPs are reported to have the intrinsic biocatalytic activity akin to horseradish peroxidase (HRP) 201. Therefore, inorganic nanoparticles have been used as nanozyme for specific biomedical applications 202, 203. The mechanism of Fe_3_O_4_ as nanozyme in killing tumor cells can be understood as the Fenton reaction, which utilizes Fe^3+^/Fe^2+^ ions reaction with excessive H_2_O_2_ in tumor tissues to generate excessive reactive oxygen species (ROS) 204-206:

*Fe^3+^ + H_2_O_2_ = Fe^2+^ + HO_2_•+ H^+^*

*Fe^2+^ + H_2_O_2_ = Fe^3+^ + •OH + OH^-^*

Due to the chain reaction between Fe_3_O_4_ (Fe^2+^, Fe^3+^) and H_2_O_2_, the Fenton reaction consumes the H_2_O_2_ in the tumor tissues and produce the •OH with cytotoxicity 207. Based on the excellent enzyme-like activities, Fe_3_O_4_ NPs have been developed as enzyme mimetics for many novel biomedical applications 201. It is worth mentioning that the efficient Fenton reaction usually require specific conditions, such as lower pH (3.0 ~ 4.0), higher temperature or UV/vis light irradiation. However, the pH value of tumor microenvironment is around 5.5-6.5, which cannot achieve the optimal Fenton reaction conditions 208. Therefore, for achieve the best therapeutic effect, the UV/vis light or thermotherapy are always used to assist the Fenton reaction to enhance the ability of producing •OH 209.

**Figure 13:**
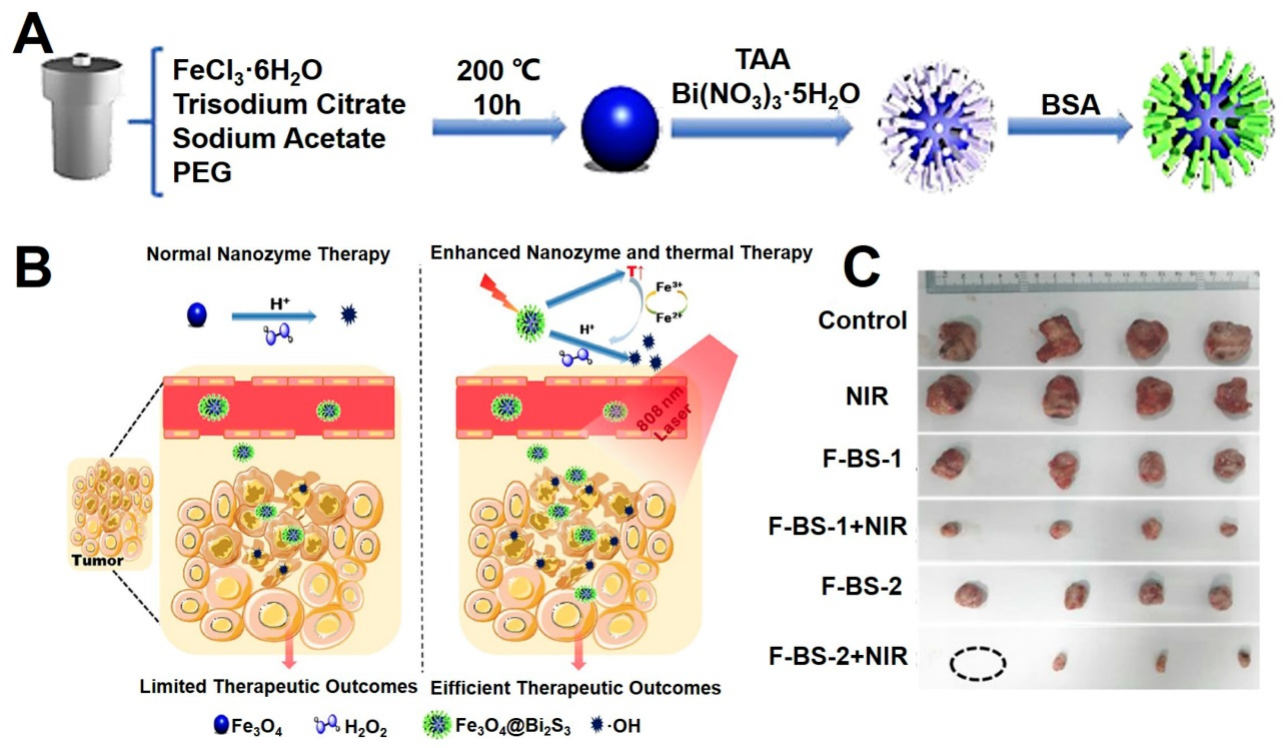
(A) The synthesis mechanism diagram of Fe_3_O_4_@Bi_2_S_3_ nanocatalysts. (B) The schematic diagram of* in vivo* photothermal and chemodynamic therapy. (C) The photographs of anatomical tumors* in vivo* of treating groups. Adapted with permission from 211, copyright 2020 ACS Applied Materials & Interfaces.

Gu *et al.* 210 verified the enzyme-like activities of Fe_3_O_4_ NPs on role position and pH values. When the Fe_3_O_4_ NPs were internalized into tumor cells mainly concentrated in lysosomes and the cytotoxicity is related to the concentration. Fe_3_O_4_ show stronger toxic potency than γ-Fe_2_O_3_. Due to the acidic microenvironment of lysosomes, Fe_3_O_4_ NPs presented the peroxidase-like activity and the cell damage would be enhanced by the induced H_2_O_2_. In neutral pH conditions, both Fe_3_O_4_ and γ-Fe_2_O_3_ NPs cannot produce hydroxyl radicals (•OH), but just catalyze decomposition of H_2_O_2_ into H_2_O and O_2_ directly. These results show that the cytotoxicity of Fe_3_O_4_ nanozyme are decided by the external environment and distribution intracellular. Lin *et al.* 211 reported a strategy for enhanced the chemodynamic therapy (CDT) effect by the synergistic of photothermal therapy, which combined the typical Fe_3_O_4_ nanozyme and the semiconductor Bi_2_S_3_ as shown in Figure 13A. The Fe_3_O_4_@Bi_2_S_3_ nanocatalysts could kill the cancer cells through the effect of photothermal treatment under 808 nm laser, and sequential thermal effect enhanced the Fenton action of Fe_3_O_4_ NPs, which could efficiently convert H_2_O_2_ into highly toxic •OH, thus realizing a remarkable synergistic anticancer achievement.

**Figure 14:**
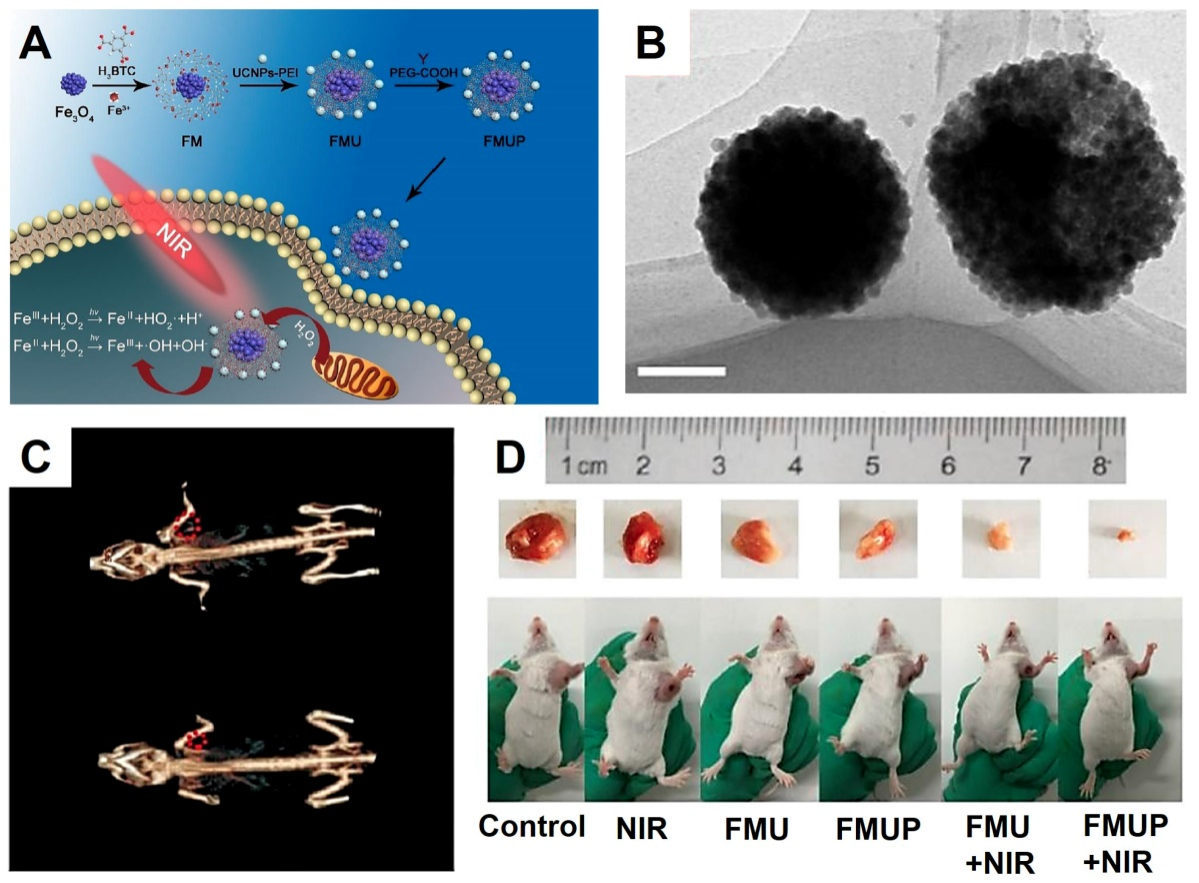
The synthesis process illustration (A) and the TEM images (B) of FMUP (Scale bars: 100 nm). (C) *In vivo* CT images of FMUP before (down) and after injection (up). (D) The photographs of tumor-bearing mice after 14 days of treatment. Adapted with permission from 209, copyright 2018 Chemical Engineering Journal.

Yang *et al.* 209 designed a novel diagnosis and treatment platform composed of Fe_3_O_4_ NPs, metal organic frame MIL-100(Fe), UCNPs (*NaYF_4_:Yb, Tm@NaGdF_4_:Yb*) and modified by PEG (aliased as FMUP). The UCNPs were designed for converter the near-infrared light to UV/vis light, which could excite the Fe^3+^/Fe^2+^ ions in the Fe_3_O_4_ NPs and enhance the effect of photo-Fenton reaction, thus activating the photocatalytic reaction by using MIL-100(Fe) as photosensitizer (Figure 14), which synergy combined photodynamic therapy (PDT) and photochemotherapy (PCT). The Fenton reaction produced cytotoxic reactive oxygen (•OH) with high cytotoxicity independent of the oxygen in the tumor microenvironment. Meanwhile, the Fe_3_O_4_ NPs and MIL-100(Fe) could form the heterojunctions that markedly inhibited the recombination of electrons and holes, thus effect enhancing the ability of PCT and PDT in producing ROS. The antitumor effect was showed by* in vivo* and* in vitro* assays, the experimental results have indicated that the FMUP with the excellent imaging ability of CT and UCL with positive synergistic treatment effect of PDT and PCT.

### 5.2 Magnetic hyperthermia treatment

Magnetic nanoparticles (MNPs) provide an effective platform for biomedicine with lots of applications such as MRI, magnetic guidance delivery, magnetic separation, and thermal treatments 212, 213. The magnetic hyperthermia treatment (MHT) is a classical therapeutic concept based the principle of cancer cells are more vulnerable than healthy cells when the environment temperatures higher than 41 ℃. The thermal effect of MNPs could generate by the external alternating magnetic field (AMF) and define by the specific absorption rate (SAR), which is rate value of energy absorbed per unit mass of the agent when located in radio frequency. The values of SAR are influenced by the morphology and composition of the MHT agent, also be affected by the property of magnetic field such as frequency (*f*) and amplitude (*H*). Therefore, for achieving an effect of hyperthermia to tumors, the magnetically mediated hyperthermia nanomaterials must show the high SAR and low (*f*)/(*H*) in the low content. Thereinto, the superparamagnetic Fe_3_O_4_ NPs as the heat mediators *via* an oscillating magnetic field show remarkable superiority, because SAR values of superparamagnetic nanoparticles can be enhanced by increasing either (*f*) or (*H*) (or both increase) during the measurements 96. In general, most studies on the SAR carried out on Fe_3_O_4_ NPs were prepared by co-precipitation method or sol-gel, the resulting nanoparticles were usually in the size of 20-50 nm. Only few researches of SAR prepared by thermal decomposition methods have reported, because small size Fe_3_O_4_ NPs usually has a low SAR value 212.

Ishimura* et al.* 214 reported the research of superparamagnetic Fe_3_O_4_ NPs (SPIONs) clusters for MRI and MHT. Superparamagnetism of Fe_3_O_4_ NPs only present in the size less than 10 nm. However, this size is smaller than capillaries pores of normal tissues and will lead to the leakage of SPIONs from the normal tissues, resulting the low accumulation in tumors, and reduce the effect of the tumor diagnosis and treatment. To obtain effective accumulation and excellent therapeutic effect of magnetic hyperthermia treatment, the FA and PEG modified SPIONs nanoclusters were designed (FA-PEG-SPION NCs). The SPIONs clusters not only prevented the leakage from normal tissues capillaries, but also increased the rate of relaxivity and the specific absorption. Meanwhile, the FA and PEG could also increase the targeting of SPIONs clusters and enhance the amount accumulation in tumors. After intravenous injection the FA-PEG-SPION NCs for 24 h, the clusters would accumulate locally in cancer (not necrotic) tissues and enhance the MRI intensity. Furthermore, the FA-PEG-SPION showed excellence magnetic hyperthermia effect under the alternating current magnetic field (*f* = 230 kHz, *H* = 8 kA/m).

**Figure 15:**
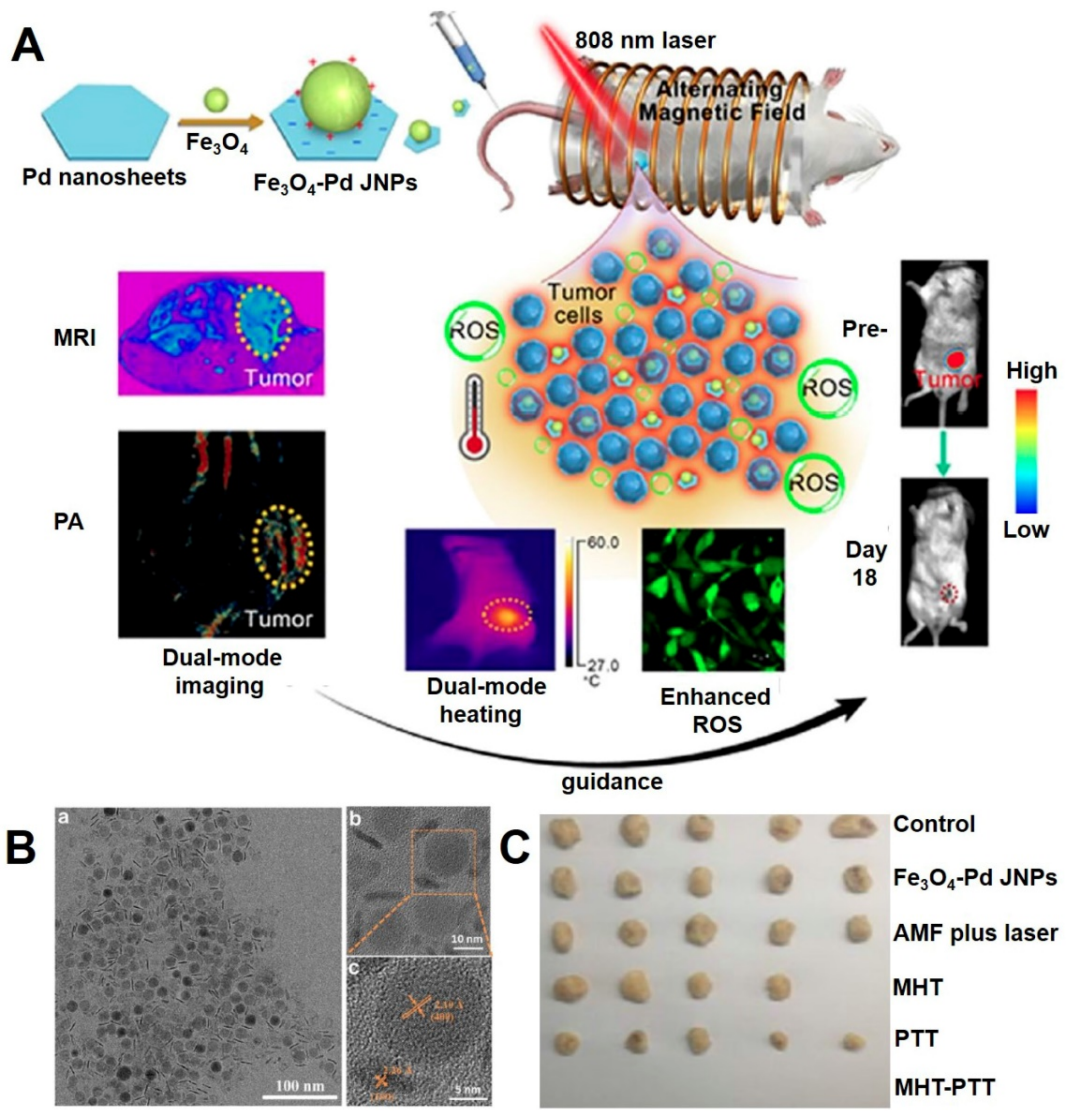
(A) The illustration of the design Fe_3_O_4_-Pd JNPs. (B) TEM images of Fe_3_O_4_-Pd JNPs (TEM (a), enlarged TEM (b) and HRTEM (c)). (C) The photos of tumor tissues harvested from mice at the end of treatment. Adapted with permission from 215, copyright 2019 Nanoscale Horizons.

Liang *et al.* 215 designed a multifunctional system consisting of Pd modified Fe_3_O_4_ nanoparticles (JNPs) with the property of dual-mode MRI and PA (photoacoustic) imaging, synergy of photothermal therapy, magnetic therapy and chemodynamic therapy (Figure 15). The plasmonic photothermia property of Pd nanosheets could achieve synergistic effects for enhancing the magnetic/photothermal effect of Fe_3_O_4_ NPs, and the chemodynamic therapy could be attributed to the •OH, which generate from the Fenton reaction of Fe_3_O_4_ NPs and catalytic properties of Pd nanosheet in an acidic environment of tumor H_2_O_2_. Meanwhile, the ability of reactive oxygen species production could also be further enhanced under alternating magnetic field and near-infrared light irradiation.

### 5.3 Photothermal therapy

Photothermal therapy (PTT) is widely used for tumor therapy in recent years, relying on the nanoparticles in tumor tissues to convert optical energy into thermal energy to kill cancer cells 216. Compared with surgical management and radiotherapy chemotherapy, PTT is more controllable, highly efficient and lower invasive 217. This treatment takes the advantage of near-infrared light (NIR) with excellent tissue penetration effect and minimum damage in the range of 700-1100 nm, which could activate specific materials to transformation energy generate thermal effect and cause damage to the tumor tissues. Many nanomaterials have been developed as PTT agents, such as noble metals, semiconductors, and special polymers, which usually have a strong optical absorbance in the near-infrared optical window 218. The potential toxicity caused by photothermal agents is still an unresolved problem because these nanoparticles tend to accumulate in organs and the slow degradability of nanomaterials would introduce inflammatory cytokine production, increase oxidative stress and cell death, which would limit the clinical application of PTT 219, 220. Therefore, that necessary to explore the agent with biosafe and biodegradable for PTT.

Fe_3_O_4_ NPs have been attention due to the excellent biocompatibility, nontoxicity, MRI and magnetic targeting capability 221. Although the Fe_3_O_4_ have been approved as drugs for clinical. However, there are few reports about Fe_3_O_4_ NPs in photothermal therapy, due to the low molar extinction coefficient and poor photothermal conversion efficiency 222. In order to improve the photothermal therapy effect of Fe_3_O_4_ NPs, much research has been done, one common way is to decorate the materials with high photothermal conversion (e.g., noble metals, copper chalcogenides, special polymer) onto the surface of magnetic Fe_3_O_4_ NPs to enhance the plasma resonance, another is to change the morphology of Fe_3_O_4_ NPs for obtaining better photothermal conversion effect 223. Shi *et al.* 224 reported the photothermal effect of special shape magnetic Fe_3_O_4_ NPs both *in vitro* and* in vivo* experiments. The heating effect of Fe_3_O_4_ NPs with the shape of spherical, hexagonal and wire-like were found rapidly generated under the red and near-infrared range laser irradiation, which showed obviously damaged on cancer cell cellular organelles. Due to the photothermal effect of the special-shaped Fe_3_O_4_ NPs, the tumor cells were found to be significant apoptosis with tumor reduction.

**Figure 16:**
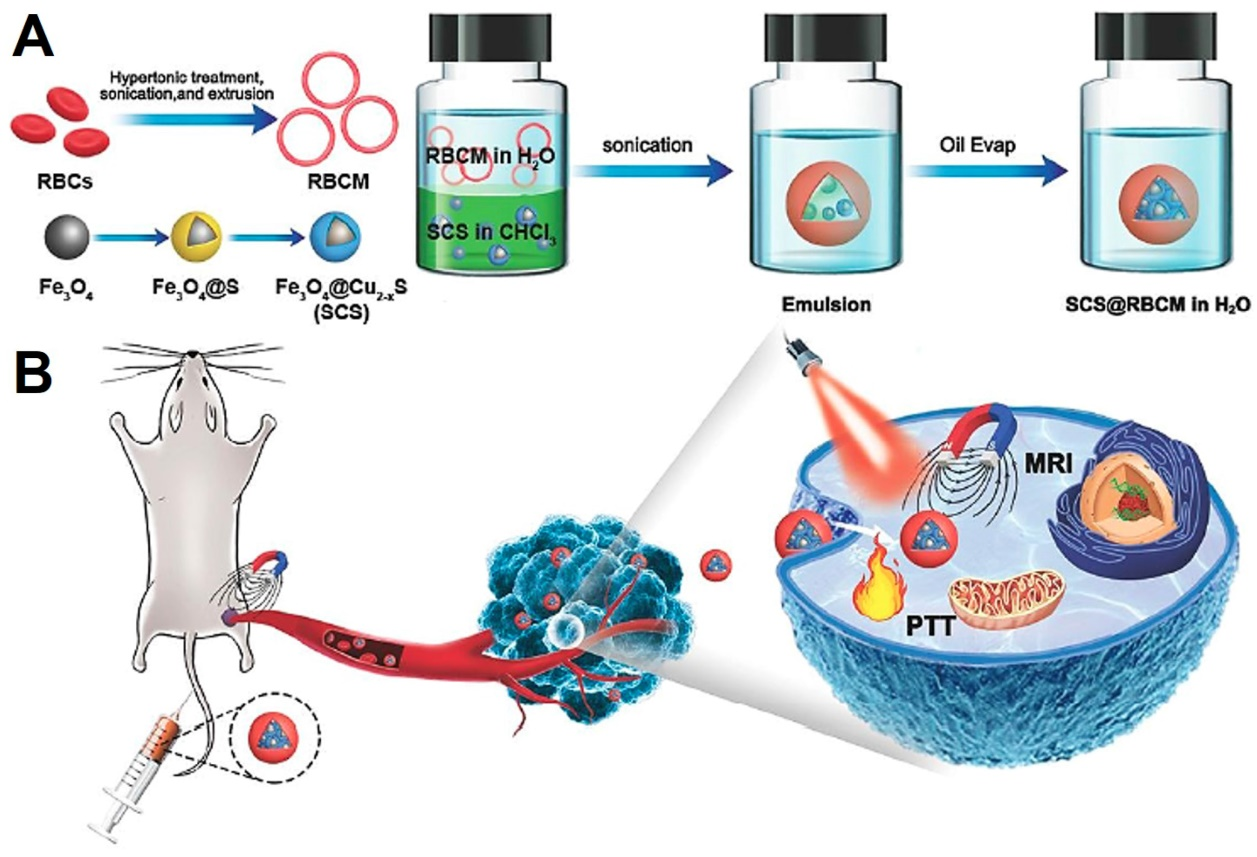
Schematic illustration showing the synthesis process of red blood cell membrane coated Fe_3_O_4_@Cu_2-x_S nanocluster system (A) and magnetic field triggered active targeting of MRI and PTT (B). Adapted with permission from 222, copyright 2020 Journal of Materials Chemistry B.

Wu *et al.* 222 proposed a simple method to transfer hydrophobic Fe_3_O_4_@Cu_2-x_S NPs to hydrophilic with the help of red blood cell membrane (Figure 16), which showed excellent performance for *T*_2_-weighted MRI and PTT. The obtained nanoplatform was consist of the densely Fe_3_O_4_@Cu_2-x_S nanocluster core and the red blood cell membrane layer shell. This system displayed a stable nanostructure, excellent magnetic targeting ability and photothermal conversion ability. With the advantages of the red blood cell membrane, the nanocluster was protected from the elimination by macrophages, and showed excellence magnetic targeting under the external magnetic field. Therefore, all of these features promote the platform with high performance for MRI and PTT.

**Figure 17:**
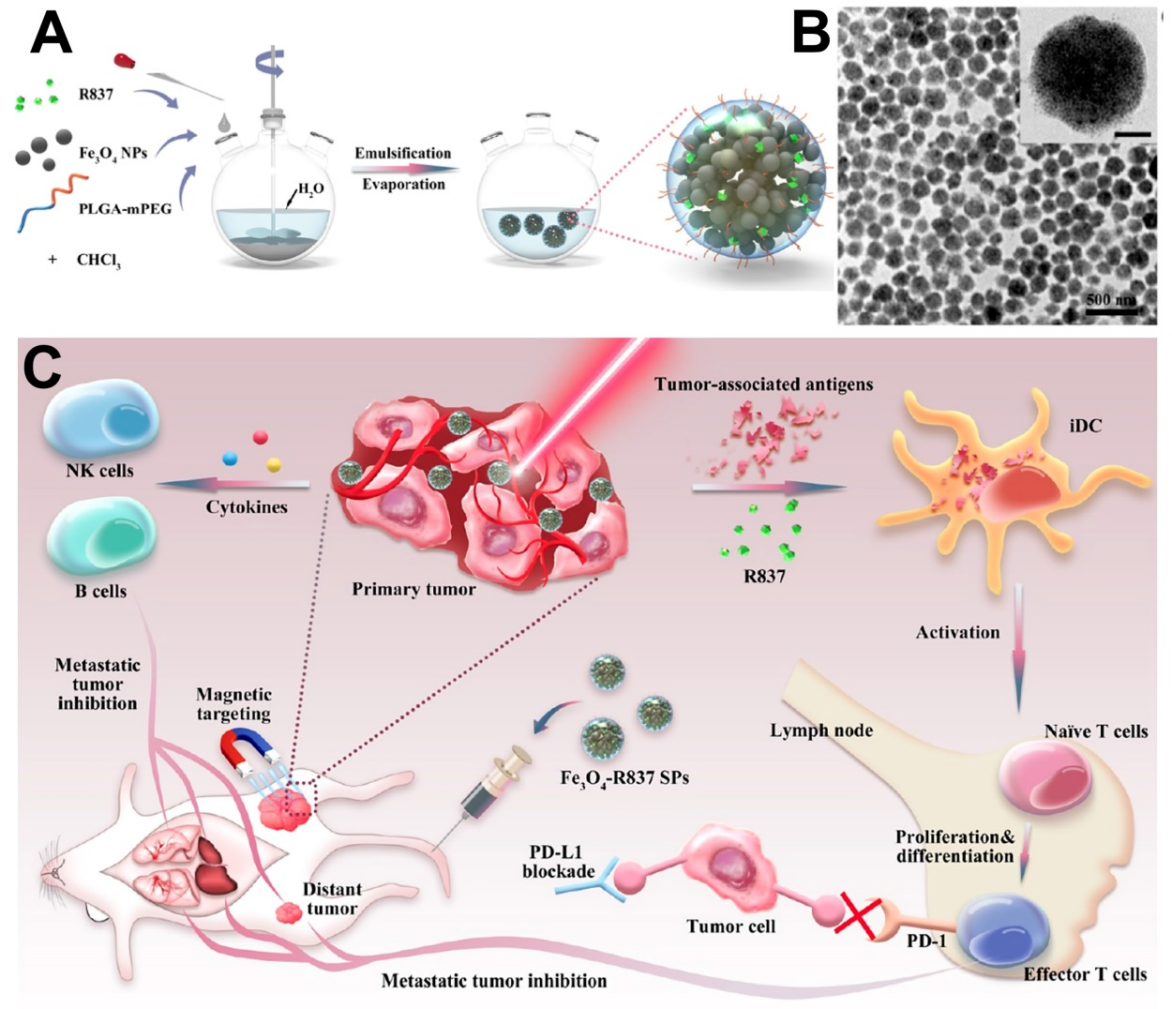
(A) The schematic illustrates of the Fe_3_O_4_-R837 SPs synthesis process. (B) TEM images of Fe_3_O_4_-R837 SPs (scale bar: 50 nm). (C) The schematic diagram of Fe_3_O_4_-R837 SPs for cancer therapy by antitumor immune responses synergistic photothermal therapy. Adapted with permission from 227, copyright 2018 ACS applied materials & interfaces.

Recent researches have demonstrated the magnetic moment could significantly enhance with the agglomeration state 225. Therefore, change the monodisperse magnetic Fe_3_O_4_ NPs into clusters provided an effective way to enhance the specific absorption rate (SAR) 226. Yang *et al.* 227 reported an efficient PTT platform by self-assembly the monodisperse Fe_3_O_4_ nanocrystal into spherical superparticles (SPs), which could significantly increase the effect of photothermal treatment (Figure 17). For further enhance the tumor treatment effect, mPEG-PLGA copolymer were used to modify the Fe_3_O_4_ SPs, and immune adjuvant R837 were loaded to generate the antigens associated to tumor and induce strong antitumor immune responses synergistic treatment of tumor.

### 5.4 Photodynamic therapy

Photodynamic therapy (PDT) is considered as an emerging therapeutic strategy for clinical anticancer in recent years, due to the low systemic toxicity, cooperativity and negligible side effects. It includes three important parts: photosensitizers (PS), exciting light and oxygen in the tissue 200. The principle of PDT is to trigger the reactive oxygen species (ROS) with cytotoxicity to induce tumor cells death. Meanwhile, the accurate delivery of photosensitizer to tumor tissue also play an important part of the treatment process. Magnetic targeting is a classic targeting approach, which takes the advantages of magnetic nanoparticles with the capability of being magnetized and directional movement by an external magnetic field, finally guidance the magnetic nanoparticles concentrate in specific location. This physical interaction exhibiting potential application for tumor targeting, and as an excellent magnetic carrier, the Fe_3_O_4_ NPs have been concerns on the excellent MRI performance, magnetic targeted ability, high biocompatibility and chemical stability. Therefore, the effective combination of photosensitizer with Fe_3_O_4_ NPs could construct the photodynamic therapy nanoplatform with the magnetic targeted and MRI guided.

**Figure 18:**
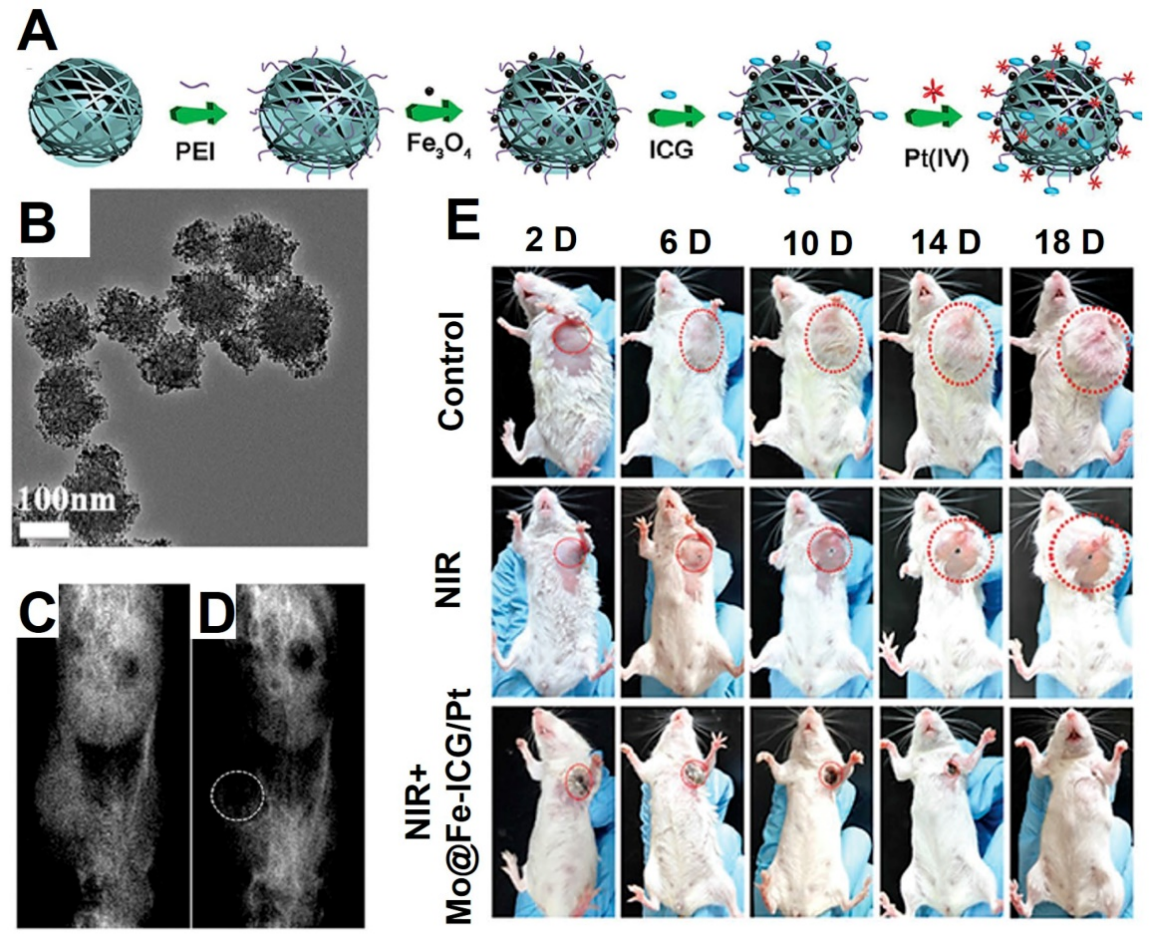
The schematic diagram of the synthesis process (A) and TEM images (B) of Mo@Fe-ICG/Pt nanocomposites. The *T*_2_-weighted MRI preinjection (C) and after injection (D) of Mo@Fe-ICG. (D) The photographs of mice after treatments by Mo@Fe-ICG/Pt. Adapted with permission from 228, copyright 2017 Advanced Science.

Lin et al. 228 synthesized a multifunctional treatment platform by covalently grafting Fe_3_O_4_ on the surface of MoS_2_, then the photosensitizers indocyanine green (ICG) molecules and prodrugs Pt (IV) were loaded on the surface of MoS_2_@Fe_3_O_4_ as shown in Figure 18A (Mo@Fe-ICG/Pt). The high transverse relaxivity Fe_3_O_4_ NPs in the Mo@Fe-ICG/Pt nanocomposites were used as an effectively *T*_2_-weighted MRI contrast agent, which provided an excellent positioning effect for photothermal therapy of MoS_2_, photodynamic therapy of ICG, and chemotherapy triggered of Pt (IV) prodrugs, thus leading to an ideal nanoplatform for tumor imaging and treatment.

**Figure 19:**
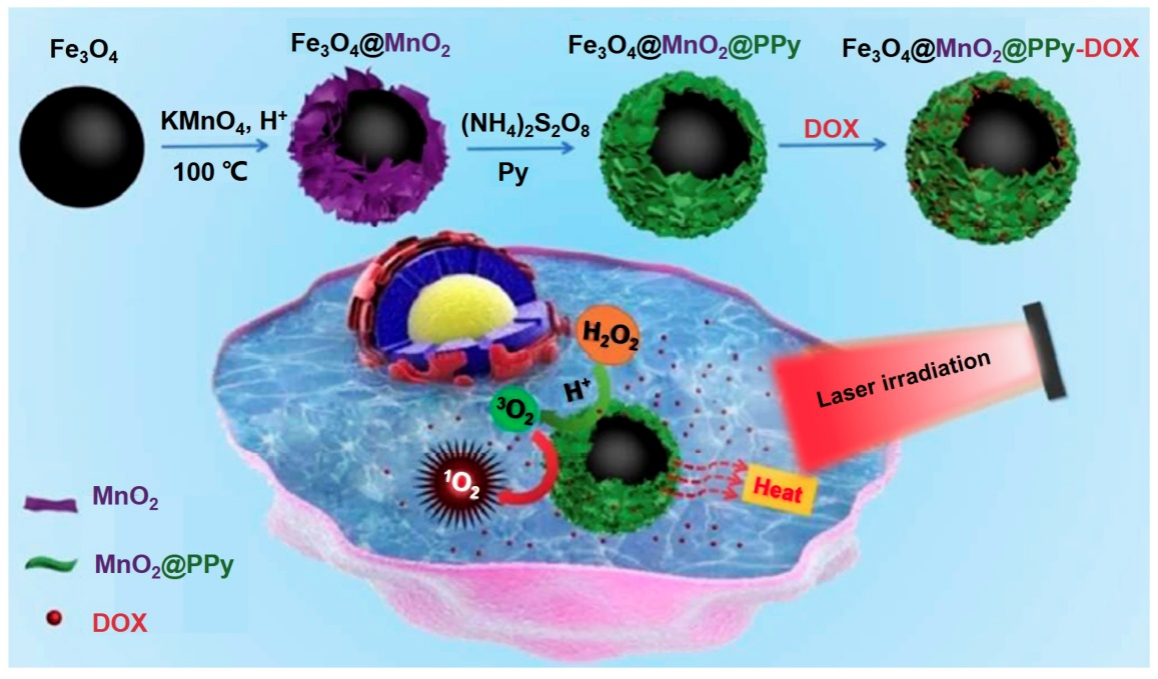
Schematic illustration for the fabrication and magnetic targeting, improved PDT by the catalytic decomposition of H_2_O_2_ and synergistic chemotherapy and PDT/PTT treatments of the Fe_3_O_4_@MnO_2_@PPy-DOX to cancer cells. Adapted with permission from 229, copyright 2018 Journal of Materials Chemistry B.

Zhu* et al.* 229 designed a multifunctional tumor treatment platform composed of Fe_3_O_4_ core and two kinds of shells, which are MnO_2_ and polypyrrole (PPy) as shown in Figure 19. The PPy was used as the agent of photothermal and photosensitizer, then combined with Fe_3_O_4_@MnO_2_ for achieving the magnetic targeting effects. The MnO_2_ shell could catalyze the H_2_O_2_ in the tumor tissues decompose into O_2_ to improve the production of ROS under the irradiation, finally enhance the therapeutic effect of PTT/PDT. Meanwhile, the Fe_3_O_4_@MnO_2_@PPy nanocomposite could also load DOX efficient and implement an acid response release in tumor tissues, which also realized the synergistic chemotherapy and PDT/PTT by avoiding damage to normal cells.

### 5.5 Chemotherapy drug delivery

The most common non-surgery methods for cancer treatment are chemotherapy and radiation therapy. Nowadays, there are about 80 different kinds of antitumor drugs in the clinic, and around 400 drugs are being tested in clinical trials 230, 231. Although they are effective in some cancer types, most of them lack specificity towards tumor cells 232. The normal tissues and organs will be harmed at therapeutic dosage 233. Tumor-targeting therapy is delivering some chemotherapy drugs or other antitumor bioactive compounds to cancer cells by specific carriers, with limited influence on normal healthy tissues, resulting in higher therapeutic efficiency and lower toxicity 234, 235. Meanwhile, the target delivery can be classified into three types based on the destination of the drugs, the primary target is reaching determined tissues or organs. The secondary target is reaching specific cells. The third-level target is interacting with some specific targets inside cells 236, 237. So far, tumor nano-delivery systems have attracted main attention on the size so that the drug could enrich in tumor tissues owing to the EPR effect of tumor tissues 238, 239. Meanwhile, the nano-delivery system could conjugate with some certain ligands to permits the active targeting into tumor tissues. Achieving highly specific targeting to cancer cells is the ultimate goal in cancer therapy and diagnosis 240, 241.

**Figure 20:**
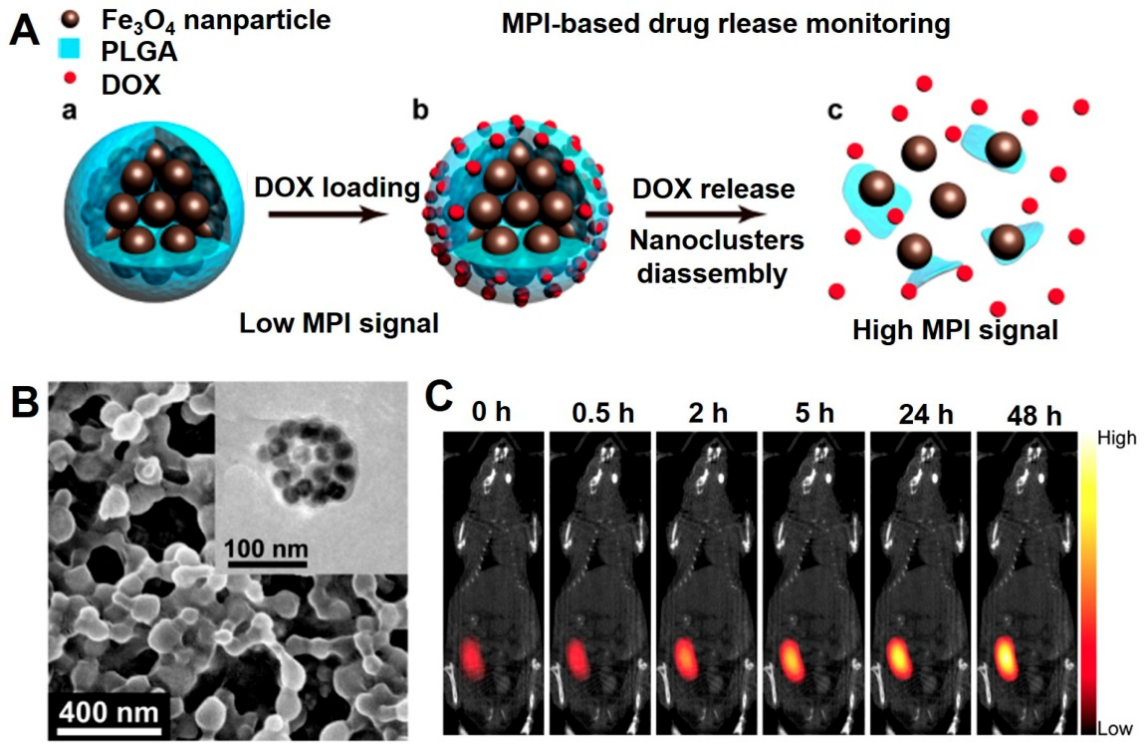
The schematic diagram of Fe_3_O_4_@PLGA nanocomposite for MPI-based drug release monitoring. (B) SEM image of DOX loaded Fe_3_O_4_@PLGA nanocomposites, and TEM image of a Fe_3_O_4_@PLGA nanocomposites (Inset). (C) MPI and CT merged images injected intratumorally with Fe_3_O_4_@PLGA. Adapted with permission from 243, copyright 2019 Nano letters.

In 1960s, Freeman* et al* has introduced the magnetically Fe_3_O_4_ NPs guided anti-cancer drugs delivery carrier is a unique method to concentrate drug accumulation of therapeutic nanoparticles to tumor tissues to improve therapeutic efficacy and used for the MRI contrast agent in 1985s 75, 242. Meanwhile, the monitoring of drug release *in vivo* provides the accurate and reliable information for guiding the chemotherapy. The *in vivo* drug delivery based on the imaging guiding shows unique advantageous due to the no invasiveness and the drug distribution visualization 243. Magnetic particle imaging (MPI) due to the deeper tissue penetration and quantifiable signal intensity are widely used in detecting drug release* in vivo*, which mainly use the superparamagnetic Fe_3_O_4_ NPs as the contrast agent and sole signal source 244, 245. Smith *et al.* 243 designed a core-shell nanocomposite consisting of the superparamagnetic Fe_3_O_4_ nanocluster and PLGA loaded with the chemotherapy drug DOX, which could be applied for drug delivery and the tracer of MPI quantification (Figure 20). The core-shell nanocomposite can occur decomposition in the tumor microenvironment (pH = 6.5), which could induce the gradual decomposition of the Fe_3_O_4_ nanocluster, sustained release of DOX and the change of MPI signal. The results indicated that induced MPI signal changes and the release rate of DOX over time displayed a linear correlation (R^2^ = 0.99). Using this phenomenon, the chemotherapy drug release process in tumor tissue can be successfully monitored to assess the induced tumor cell damage.

**Figure 21:**
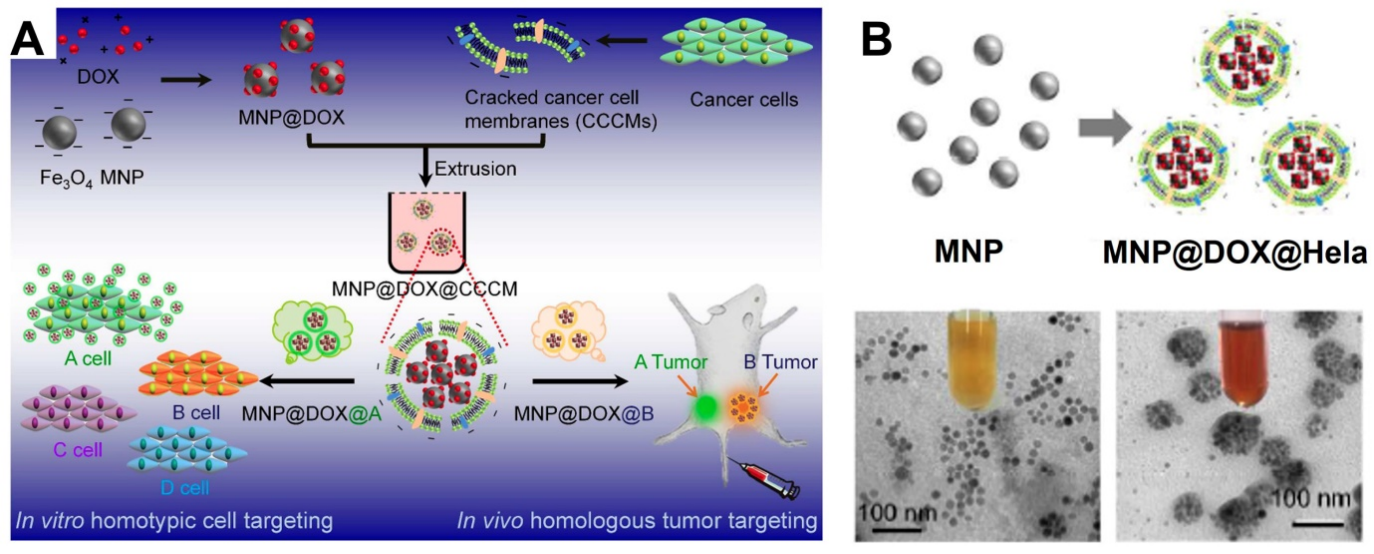
(A) The schematic diagram of cracked cancer cell membrane (CCCM) embellished MNPs and the mechanism for prioritizing cancer cells recognition; (B) TEM images of MNP and MNP@DOX@CCCM. Adapted with permission from 250, copyright 2016 Nano Letters.

In recent years, the delivery of drug using cancer cell membrane, especially that from the homologous tumors has become an emerging tumor-targeting modification method 246. In this context, a series of cancer cell membrane modified Fe_3_O_4_ NPs for the cancer diagnosis and treatment are reported. By modifying employ the source cancer cell membrane *in vitro*, the Fe_3_O_4_ NPs are identified with the self-recognition internalization, and “homing” targeting ability to the homologous tumor *in vivo* to precisely deliver the drugs to the appropriate tumor sites 247, 248. As a result, Fe_3_O_4_-based delivery system shows strong curative effect for MRI and tumor treatment *in vivo*. This delivery strategy by modifying cell membrane on the traditional delivery system have shown great potentials for precise targeting to particular tumors by merely adjusting the corresponding type of modified cell membrane 249, 250. Zhang *et al.* 250 devised a magnetic Fe_3_O_4_ NPs (MNP) based diagnosis and treatment nanomaterials platform, and modifed the cracked cancer cell membranes (CCCM) on the surface coating the Fe_3_O_4_ NPs with the particular kind of cell membrane derived from a specific delivery system to correspond type of tumor (Figure 21). Meanwhile, the chemotherapy drug DOX can also be delivered with the CCCM modified MNP system, which showed self-recognition to the same cancer cell lines by the “homing” to the homologous tumor *in vivo.* These results provide a new approach to develop highly tumor-recognizing self-targeting nanosystems for cancer therapy and diagnosis.

### 5.6 Gene delivery

**Figure 22:**
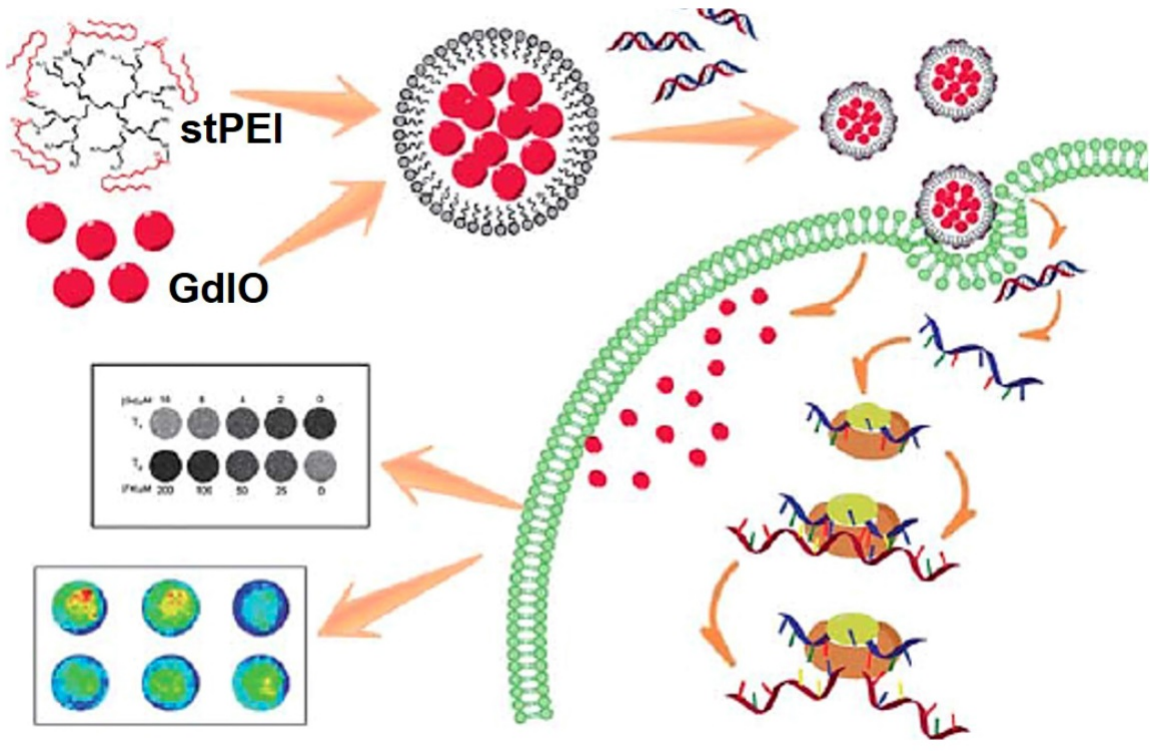
Schematic illustration of the preparation of GdIO-stPEI/siRNA complexes and their function. Adapted with permission from 259, copyright 2013 Nanoscale.

Small interfering RNA (siRNA) could downregulate specific protein expression by suppressing a targeted gene selectively at the mRNA transcription level through a mechanism called RNA interference (RNAi) 251, 252, which have been widely researched for treating a variety of genetic diseases, such as cardiovascular diseases and cancers 253, 254. Despite of these advantages, the low transport efficiency of siRNA with severe side effects still limits the clinical use 255, 256. Fe_3_O_4_ NPs are well-established depend on possess magnetic properties, actively investigated as new generation for MRI, excellent biocompatibility and versatile surface functionalization capability, due to the unique characteristics, Fe_3_O_4_ NPs are extensively explored for various biomedical applications especially in delivery of therapeutics, antibodies, peptides, nucleic acids, and other assorted biological agents biological agents 257, 258. Chen* et al.* 259 reported a multifunctional nanoclusters as an agent for dual-modal* T*_1_-*T*_2_ MRI imaging and siRNA delivery (Figure 22). The hydrophilic nanoclusters were self-assembled from the hydrophobic gadolinium (Gd) embedded Fe_3_O_4_ nanocrystals (GdIO), after self-assembly with polyethylenimine (stPEI), the obtained GdIO-stPEI nanoclusters showed good stability, dispersity and dual-modal *T*_1_-*T*_2_-weight MRI properties. Meanwhile, the composite nanoclusters with the ability to deliver the siRNA, while maintaining other properties such as magnetism, biocompatibility, and imaging performance, thus providing a safe and efficient method for imaging guidance gene delivery.

## Conclusion and Perspective

In summary, we reviewed the recent research of Fe_3_O_4_ NPs from the synthesis, surface modification, and focus description the novel biomedical applications in tumor imaging and therapy. With the development of nanotechnology more high quality Fe_3_O_4_ NPs synthesis methods are reported, and the surface modification of Fe_3_O_4_ NPs also became more functional. New-generation Fe_3_O_4_-based contrast agents could realize the multimode and responsive imaging, provided more accurate and effective information for tumor diagnosis and guided follow treatment.

At present, the clinical application of multifunctional Fe_3_O_4_ is mainly in MRI contrast agent, not really implemented the integration of diagnosis and treatment 260. First, the complex preparation process, high cost and long tumor treatment trial period limit the clinical use of Fe_3_O_4_-based drugs. Second, although the magnetic targeting could increase the accumulation of Fe_3_O_4_-based drugs in tumor tissues, the available percentage still less than 2 % of the intravenously administrated dose. Third, there may be no EPR effect of the nanomaterials in the solid tumor of human beings comparing with the nude mouse tumor models. Last but not the least important, the potential toxicity of nanoparticles has not been properly addressed. Totally, these disadvantages limit the Fe_3_O_4_-based drugs for the further clinical applications. We believe these advanced reports will guide the academic researchers and industries to accurate the present translational stage for the further bioapplications utilized with these multifunctionalized magnetic iron oxide nanomaterials.

## Figures and Tables

**Table 1:**
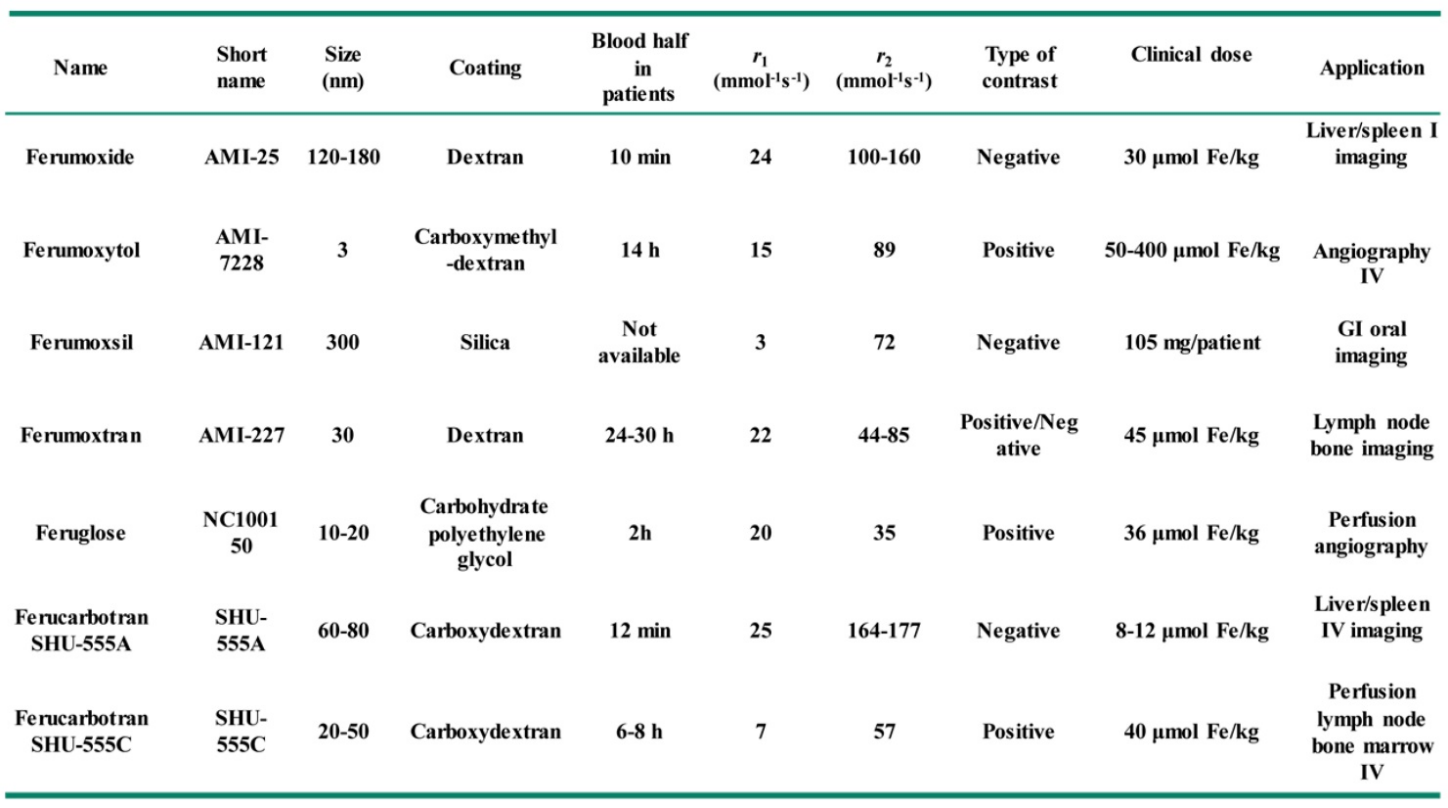
Applications of magnetic Fe_3_O_4_ NPs in clinic.

**Table 2:**
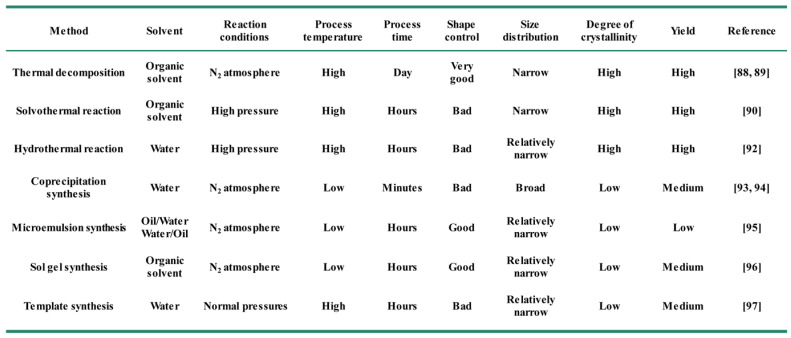
Comparison of the different chemical synthesis methods of Fe_3_O_4_ NPs.

**Table 3:**
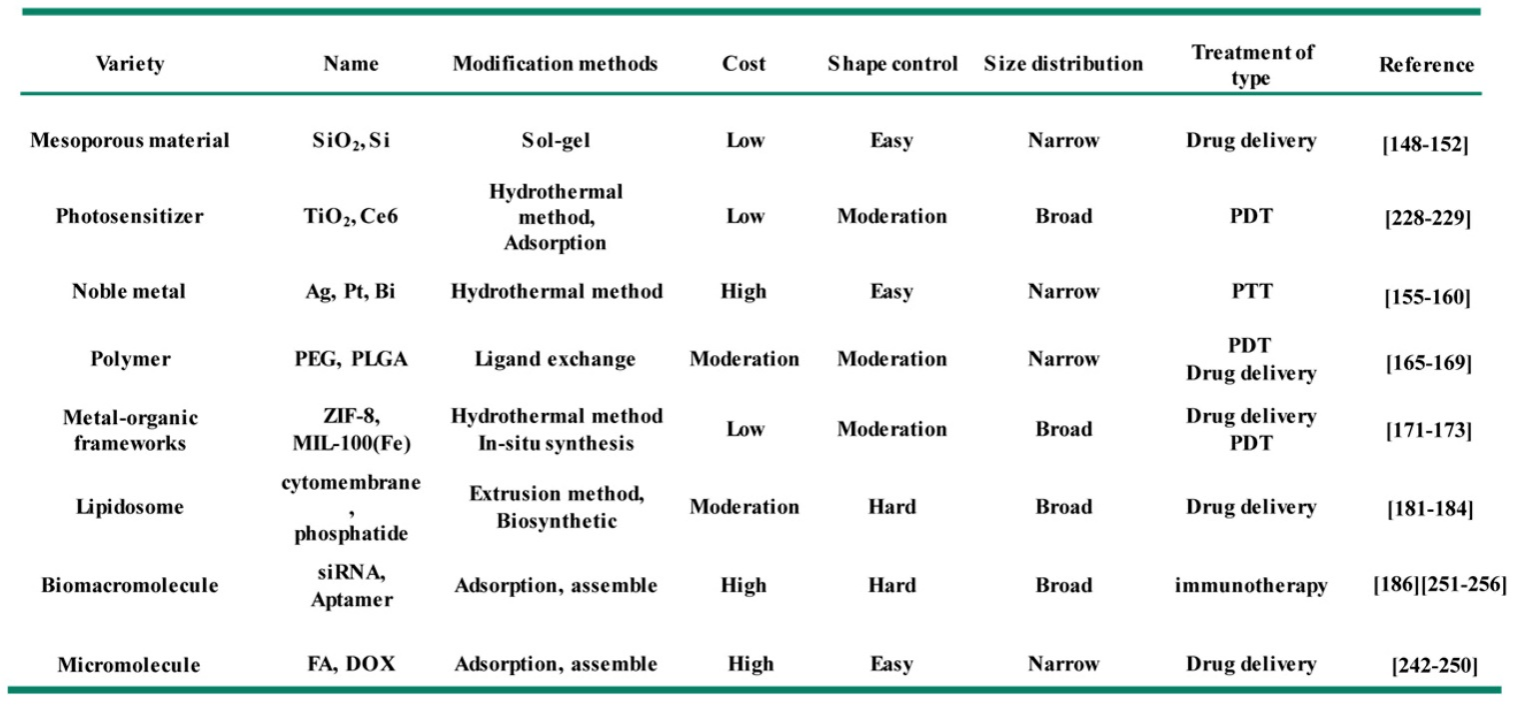
Comparison of different modification methods of Fe_3_O_4_ NPs.

## Abbreviations

MNPs): multifunctional magnetic nanoparticles
Fe_3_O_4_): magnetic iron oxide
NPs): nanoparticles
EPR): enhanced permeation and retention
SPIONs): superparamagnetic iron oxide nanoparticles
MRI): magnetic resonance imaging
TME): tumor microenvironment
Gd): gadolinium
MOF): metal-organic frameworks
PVP): polyvinylpyrrolidone
PEG): polyethylene glycol
PEI): polyethyleneimine
OA): Oleic acid
ODE): 1-octadecene
OAm): oleylamine
PLGA): poly (lactic-coglycolicacid
PS): polystyrene
PAA): polyacrylicacid
THF): tetrahydrofuran
MPTMS): 3-mercapto propyl trimeth oxysilane
PLL): poly(L-lysine
PPI): poly(propyleneimine
PGLSAOH): poly(glycerol succinic acid
PVA): poly (vinyl alcohol
MCs): microcapsules
PME): premix membrane emulsification
PLT): platelet
NIR): near infrared
PTT): photothermal therapy
SELEX): systematic evolution of ligands by exponential enrichment
DOX): doxorubicin
AG NGs): alginate nanogels
PEI): polyethyleneimine
PO): phosphine oxide
UCNPs): upconversion nanoparticles
HRP): horseradish peroxidase
ROS): reactive oxygen species
PCT): photochemotherapy
NIR): near-infrared
PDT): photodynamic therapy
CT): computed tomography
UCL): upconversion luminescence imaging
PS): photosensitizers
^1^O_2_): singlet oxygen
ICG): indocyanine green
MHT): magnetic hyperthermia treatment
SAR): specific absorption rate
SPs): superparticles
O/W): oil-in-water
DCs): dendritic cells
DLNs): draining lymph nodes
CCCM): cracked cancer cell membranes
siRNA): small interfering RNA
RNAi): RNA interference
GdIO): gadolinium embedded iron oxide
stPEI): stearic acid modified low molecular weight polyethylenimine
